# Structural Diversity of Copper(II) Complexes with *N*-(2-Pyridyl)Imidazolidin-2-Ones(Thiones) and Their *in Vitro* Antitumor Activity

**DOI:** 10.3390/molecules191017026

**Published:** 2014-10-23

**Authors:** Łukasz Balewski, Franciszek Sączewski, Patrick J. Bednarski, Maria Gdaniec, Ewa Borys, Anna Makowska

**Affiliations:** 1 Department of Chemical Technology of Drugs, Faculty of Pharmacy, Medical University of Gdańsk, 80-416 Gdańsk, Poland; E-Mails: lbalewski@gumed.edu.pl (L.B.); edziemidowicz-borys@dr-knoell-consult.com (E.B.); a.materna@gumed.edu.pl (A.M.); 2 Department of Pharmaceutical and Medicinal Chemistry, Institute of Pharmacy, University of Greifswald, L.-F.-Jahn Str., D-17489 Greifswald, Germany; E-Mail: bednarsk@uni-greifswald.de; 3 Faculty of Chemistry, A. Mickiewicz University, 60-780 Poznań, Poland; E-Mail: magdan@amu.edu.pl

**Keywords:** 1-(2-pyridyl)imidazolidin-2-ones, 1-(2-pyridyl)imidazolidine-2-thiones, copper(II) complexes, X-ray crystal structure analysis, *in vitro* antitumor activity

## Abstract

Six series of structurally different mono- and binuclear copper(II) complexes **5**–**10** were obtained by reacting *N*-(2-pyridyl)imidazolidin-2-ones (**1a**–**l**), *N*,*N*'-bis(2-pyridyl)imidazolidin-2-ones (**2a**,**b**), *N*-acyl-*N*'(2-pyridyl)imidazolodin-2-ones (**3a**–**j**) and *N*-(2-pyridyl)imidazolidine-2-thiones (**4a**–**g**) with copper(II) chloride at an ambient temperature. The coordination modes of the complexes obtained were established by elemental analysis, IR spectroscopic data and single crystal X-ray diffraction studies. The* in vitro* cytotoxic activities of both the free ligands and copper(II) complexes were evaluated using a crystal violet microtiter plate assay on five human tumor cell lines: LCLC-103H, A-427, SISO, RT-4 and DAN-G. The free ligands **1**–**4** at concentration attainable in cancer cells of 20 μM showed no meaningful cytotoxic effect with cell viability in the range of 88%–100%. The most potent copper(II) complex of 1-(6-ethoxy-2-pyridyl)imidazolidin-2-one (**6b**) exhibited selective cytotoxicity against A-427 lung cancer cell line, while the complexes of 1-(5-methyl-2-pyridyl)imidazolidine-2-thione (**5h**) and 1-(4-*tert*-butyl-2-pyridyl)imidazolidine-2-thione (**5j**) showed cytostatic effect against a whole panel of five human tumor cell lines. In conclusion, the only complexes that showed remarkably increased activity in comparison to the free ligands were those obtained from *N*-(2-pyridyl)imidazolidine-2-thiones **4c** and **4e** substituted with alkyl group at position 4 or 5 of pyridine ring.

## 1. Introduction

Among the transition metals copper occupies a unique position with respect to its biological role. Copper, which is found in living organisms, is an essential cofactor in a number of enzymes and is involved in the function of several proteins and physiological processes such as cell metabolism, mitochondrial respiration, antioxidation processes, synthesis of some active compounds [1,2]. Additionally, copper a redox-active metal may form stable complexes with chelate ligands containing donor atoms such as nitrogen, sulfur or oxygen [2].

In the field of medicinal chemistry it has been found that complexes of transient metals such as copper may possess a higher biological activity compared to the free ligands, with lower toxicity and improved physicochemical properties [3]. Moreover, coordination may lead to significant reduction of drug-resistance. Therefore a considerable research has been devoted to the synthesis of copper compounds which exhibit anticancer [3,4,5,6,7,8,9,10], SOD-mimicking [11,12,13], antimicrobial [14], anti-parasitic [15] and anti-inflammatory properties [16].

**Figure 1:**
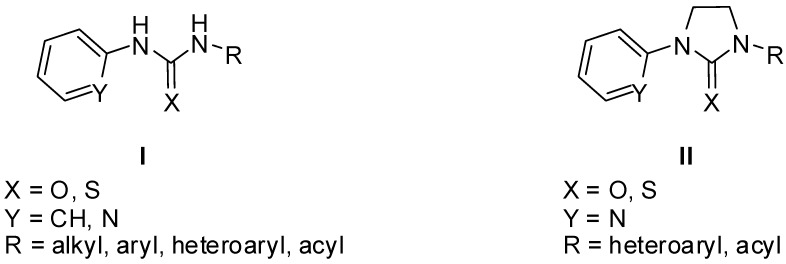
*N*-aryl(heteroaryl)ureas (**I**) and their cyclic analogues **II**.

Recently, our attention has been focused on the cyclic analogues of *N*-aryl(heteroaryl)ureas and *N*-aryl(heteroaryl)thioureas of type **I** (Figure 1) with proved anticancer activity [17,18,19]. In this paper, we wish to report the results of our studies on the synthesis and reactions of cyclic ureas and thioureas of Type **2** (Figure 1) with copper(II) chloride, X-ray structure determination of the complexes obtained, as well as the results of evaluation of their* in vitro* cytotoxic activity against several human tumor cell lines.

## 2. Results and Discussion

### 2.1. Synthesis of Ligands

**Scheme 1:**
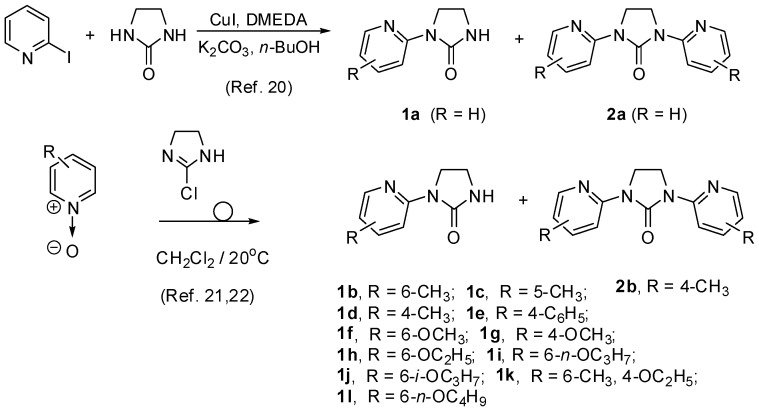
Synthesis of bidentate ligands **1a**–**l** and tridentate ligands **2a**–**b**.

Two series of chelating ligands **1a**–**l** and **2a**–**b** with N, O or S donor atoms are shown in Scheme 1. The bidentate ligand **1a** and tridentate ligand **2a** were prepared by copper-catalyzed N-heteroarylation of 2-imidazolidinone,* i.e.*, by reacting 2-imidazolidinone with 2-iodopyridine in the presence of CuI, *N*,*N*'-dimethylethylenediamine and K_2_CO_3_ in *n*-BuOH at 100 °C [20]. The substituted ligands **1b**–**l** and **2b** were obtained according to the previously described α-ureidation of corresponding pyridine-*N*-oxides with 2-chloroimidazoline [21,22].

**Scheme 2:**
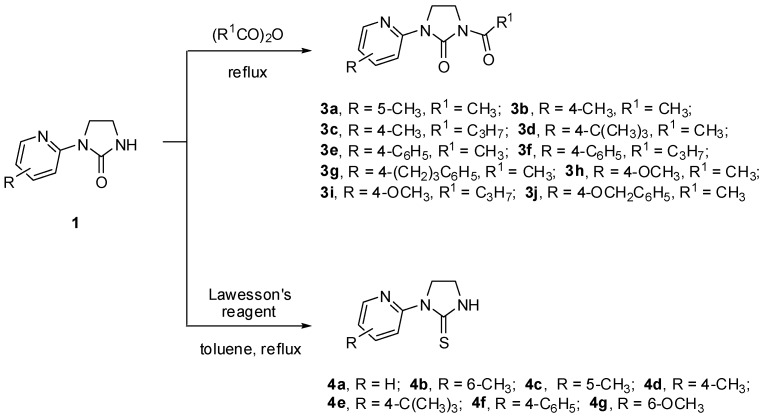
Preparation of novel *N*-acyl-imidazolidin-2-ones **3a**–**j** and imidazolidine-2-thiones **4a**–**g**.

Novel *N*-acyl-imidazolidin-2-one tridentate ligands **3a**–**j** suitable for preparation of the coordination compounds were obtained by the treatment of **1** with acetyl or butyryl anhydride, as shown in Scheme 2. On the other hand, imidazolidin-2-ones **1** were also converted into the corresponding imidazolidine-2-thiones using standard method with Lawesson’s reagent in boiling toluene (Scheme 2).

### 2.2. Synthesis and Structure of Cu(II) Complexes

**Scheme 3:**
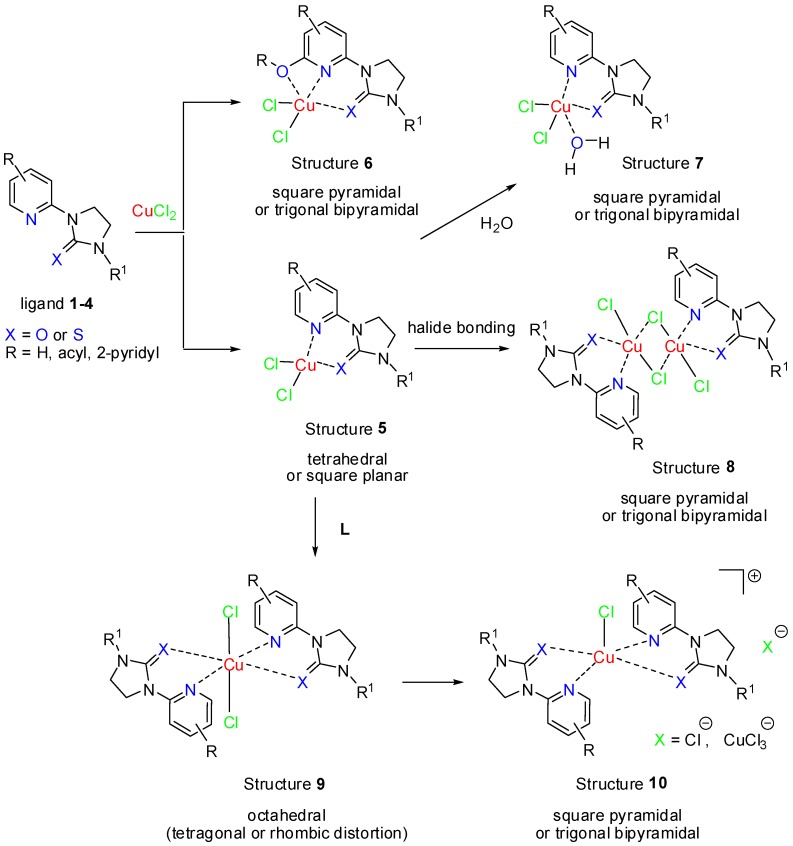
Reaction of *N*-(2-pyridyl)imidazolidin-2-one(thione) ligands **1**, **2**, **3** and **4** with CuCl_2_.

The reaction of *N*-(2-pyridyl)imidazolidin-2-one(thione) ligands **1**, **2**, **3** and **4** with CuCl_2_ were carried out at room temperature in either DMF or methanol solution containing 1% of water. Crystals suitable for the X-ray analysis were obtained by slow evaporation of the solvent. According to the X-ray data collected during the study, the following sequence of events is involved in this reaction yielding complexes with different geometries depending on the nature of ligand (Scheme 3):

(i) Initial formation of the LCuCl_2_ complex of type **5** from bidentate ligands (2-alkyl-pyridines) with tetrahedral or square planar configuration, or five-coordinate **6** from tridentate ligands (2-alkoxy-pyridines) with square pyramidal or trigonal bipyramidal configuration.
(ii) Four-coordinate complex **5** can then react with a molecule of water to give a five-coordinate complex **7**.
(iii) Complexes of type **5** can also form di-μ-chloro dinuclear five-coordinate [Cu_2_(L)_2_Cl_4_] complexes of type **8** or react with a second molecule of ligand to give octahedral [Cu(L)_2_Cl_2_] complexes of type **9**.
(iv) A geometrical change occurs upon dissociation of a weakly bonded axially coordinated chloride anion from **9**, leading to square pyramidal complexes **10** with the same *sp*^3^*d*^2^ electronic geometry.

It should be pointed out, that the preferential formation of a particular complex type may depend on solubility of **5**,* i.e.*, precipitation of **5** prevents subsequent formation of **7**, **8**, **9** and **10**. It is also possible that several species are in equilibrium in solution, and which are obtained in crystalline form depends on solubility, crystallization kinetics and other, medium dependent, properties. List of ligands **1**–**4** and corresponding complexes **5**–**10** obtained is presented in Table 1.

**Table 1:** List of ligands and copper(II) complexes obtained.

Ligand				Complex ^†^					
No	X	R	R^1^	5	6	7	8	9	10
**1a**	O	H	H	5a					
**1b**	O	6-CH_3_	H	**5b**					
**1c**	O	5-CH_3_	H					**9**	
**1d**	O	4-CH_3_	H	5c					
**1e**	O	4-C_6_H_5_	H						**10a**, **10b**
**1f**	O	6-OCH_3_	H		**6a**				
**1g**	O	4-OCH_3_	H	5d					**10c**
**1h**	O	6-OC_2_H_5_	H		6b				
**1i**	O	6-*n*-OC_3_H_7_	H		6c				
**1j**	O	6-*i*-OC_3_H_7_	H		6d				
**1k**	O	6-CH_3_, 4-OC_2_H_5_	H	5e					
**1l**	O	6-*n*-OC_4_H_9_	H		6e				
**2a**	O	H	2-pyridyl				**8a**		
**2b**	O	4-CH_3_	2-pyridyl			**7**			
**3a**	O	5-CH_3_	COCH_3_				8b		
**3b**	O	4-CH_3_	COCH_3_				**8c**		
**3c**	O	4-CH_3_	COC_3_H_7_				8d		
**3d**	O	4-C(CH_3_)_3_	COCH_3_				8e		
**3e**	O	4-C_6_H_5_	COCH_3_				8f		
**3f**	O	4-C_6_H_5_	COC_3_H_7_				8g		
**3g**	O	4-(CH_2_)_3_C_6_H_5_	COCH_3_				8h		
**3h**	O	4-OCH_3_	COCH_3_				8i		
**3i**	O	4-OCH_3_	COC_3_H_7_				8j		
**3j**	O	4-OCH_2_C_6_H_5_	COCH_3_				8k		
**4a**	S	H	H	5f					
**4b**	S	6-CH_3_	H	5g					
**4c**	S	5-CH_3_	H	**5h**					
4d *	S	4-CH_3_	H	**5i**					
**4e**	S	4-C(CH_3_)_3_	H	**5j**					
**4f**	S	4-C_6_H_5_	H	5k					
**4g**	S	6-OCH_3_	H		6f				

Notes: **^†^** Numbers in bold denote complexes whose structure was confirmed by X-ray analysis; * Ligand prepared according to ref. [21].

Copper(II) complexes exhibit a variety of irregular stereochemistries as a result of the lack of spherical symmetry of this *d*^9^ ion. Classification of coordination geometry of the obtained complexes was accomplished based on the equation described by D. Venkataraman and co-workers (Equation (1)), which determines the best fit of the observed structure of complex compound to the ideal coordination polyhedra [23]. The best fit shows minimum of deviation in ligand-copper-ligand bond angles (<L-Cu-L) between the observed coordination structure and the reference polyhedra with the same coordination number (CN). Such classification is unambiguous since a unique set of angles exists for each of the reference polyhedral. Hence, the coordination geometry is classified as polyhedron that gives the smallest value of the average angular displacement (ΔΘ) according to the following Equation (1):

ΔΘ = Σ_*i*=1_^(*n*/2) x (*n*−1)^)|Θ_i_ − Θ°_i_|/*n*/2 × (*n* − 1) (1)

where: *n*—coordination number, Θ_i_—the angle of the observed structure, Θ°_i_—corresponding valence angle of the reference polyhedron under consideration, ΔΘ—evaluation of the average angular displacement.

**Figure 2:**
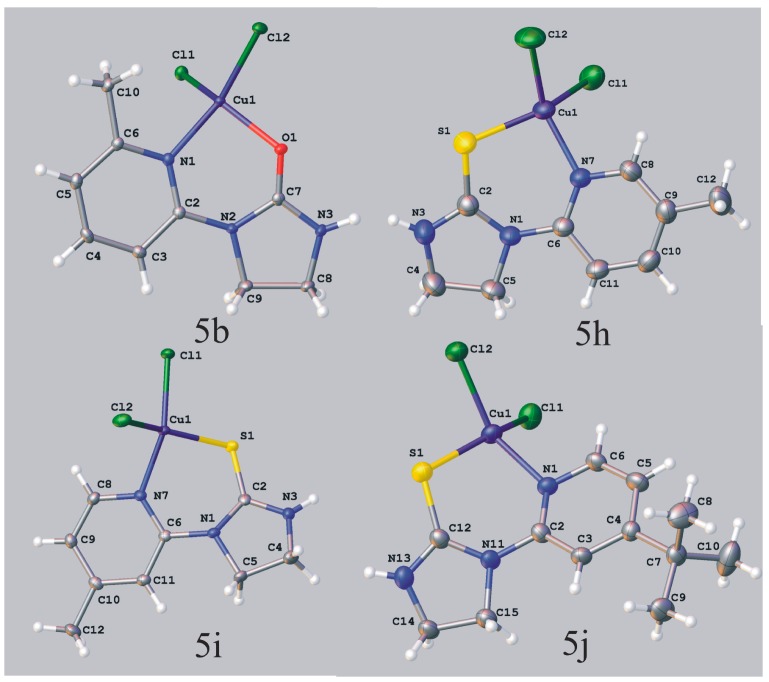
ORTEP [24] representation of molecular structure of **5b**, **5h**, **5i** and **5j**.

For example, in the case of a complex **5b** with coordination number n = 4, the number of valence angles < L-Cu-L, according to the formula n = (n/2) × (n − 1), is 6. The geometry of this complex may be: square planar with ideal angles of 90, 90, 90, 90, 180, 180 (°) or tetrahedral with angles 109.5, 109.5, 109.5, 109.5, 109.5, 109.5 (°). Comparison of the observed angles in the structure of **5b** (Figure 2, Table 2) with ideal values of each polyhedron geometry angles determined by the value of (X *vs.* Y *vs.* Z) indicates that the coordination geometry in CuNOCl_2_ core is intermediate between square-planar and tetrahedral, however, the copper ion adopts geometry that fit best to the square planar (ΔΘ = 17.79 for square planar vs ΔΘ = 18.72 for tetrahedral geometry).

A similar distorted square planar coordination geometries were found in crystals of imidazolidine-2-thiones **5h**, **5i** and **5j** (Figure 2, Table 2).

**Table 2:** Selected bond lengths and bond angles in copper complexes **5b**, **5h**, **5i** and **5j**.

No.	Bond Lengths (Å)		Bond Angles (°)	
**5b**	Cu1-Cl1	2.2239(5)	Cl1-Cu1-N1	98.45(5)
	Cu1-Cl2	2.2096(5)	Cl1-Cu1-Cl2	103.57(2)
	Cu1-N1	1.977(2)	Cl1-Cu1-O1	135.46(4)
	Cu1-O2	1.980(1)	O1-Cu1-Cl2	93.53(4)
			Cl2-Cu1-N1	143.81(6)
			O1-Cu1-N1	90.43(7)
**5h**	Cu1-Cl1	2.261(1)	Cl1-Cu1-Cl2	100.99(3)
	Cu1-Cl2	2.2139(6)	Cl1-Cu1-S1	141.86(3)
	Cu1-N7	1.977(1)	Cl1-Cu1-N7	97.01(6)
	Cu1-S1	2.2324(8)	Cl2-Cu1-N7	139.51(6)
			Cl2-Cu1-S1	93.25(3)
			S1-Cu1-N7	94.50(6)
**5i**	Cu1-Cl1	2.239(3)	Cl1-Cu1-Cl2	97.44(2)
	Cu1-Cl2	2.239(4)	Cl1-Cu1-S1	91.17(2)
	Cu1-N7	1.983(3)	Cl2-Cu1-N7	94.33(4)
	Cu1-S1	2.2708(8)	Cl1-Cu1-N7	149.94(4)
			Cl2-Cu1-S1	152.71(2)
			N7-Cu1-S1	90.88(4)
**5j**	Cu1-Cl1	2.239(3)	Cl1-Cu1-Cl2	96.92(1)
	Cu1-Cl2	2.243(3)	Cl2-Cu1-S1	90.85(9)
	Cu1-N1	2.011(6)	Cl1-Cu1-S1	146.47(9)
	Cu1-S1	2.245(2)	Cl2-Cu1-N1	143.0(2)
			Cl1-Cu1-N1	96.94(1)
			Cl2-Cu1-S1	90.87(9)

**Figure 3:**
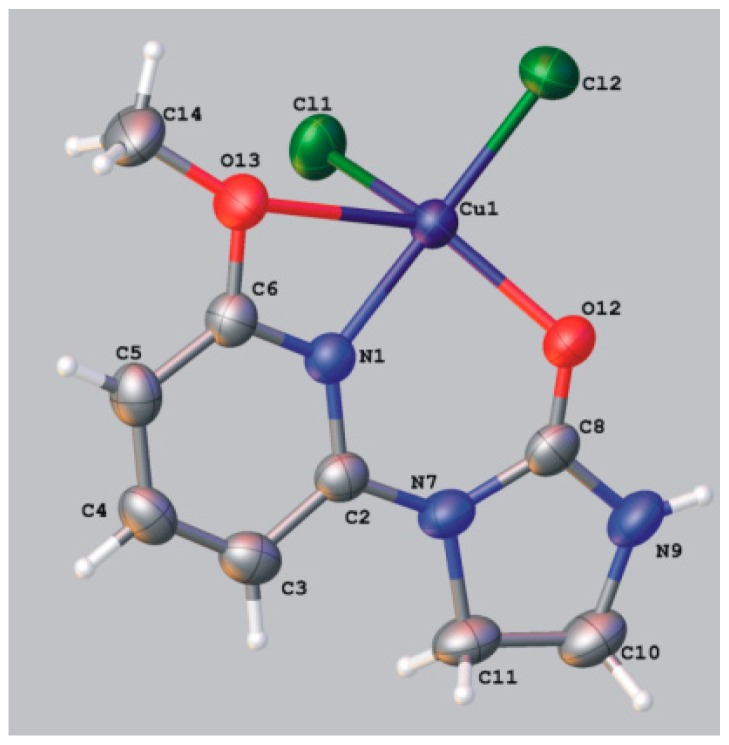
ORTEP representation of molecular structure of **6a**.

Ligands **1** and **4** containing alkoxy group at position 6 of pyridine ring form mononuclear five-coordinate (4 + 1) copper(II) complexes with the central atom chelated by neutral ligand and bound to two chloride ions and oxygen of alkoxy group. In the complex **6a** both pyridine and imidazolidine rings are approximately planar with N1-C2-N7-C8 torsion angle of 14.4°. As exemplified by the crystal structure of **6a** (Figure 3, Table 3), the geometry around Cu(II) is best described as distorted trigonal bipyramid. Atoms -Cu1-N1-C2-C8-N7-O12- form six-membered ring and atoms -Cu1-N1-C6-O13- form four-membered ring of considerable tension. The length of the bond between the atoms Cu1 and O13 of the methoxy group is longer (2.647 Å) than that between Cu1 atom and the oxygen atom O12 of the carbonyl group (1.994 Å).

**Table 3:** Selected bond lengths and bond angles in copper complex **6a**.

Bond Lengths (Å)		Bond Angles (°)	
Cu1-Cl1	2.2325(2)	Cl1-Cu1-Cl2	104.23
Cu1-Cl2	2.1848(2)	N1-Cu1-Cl2	147.16
Cu1-O12	1.9943(2)	O12-Cu1-Cl2	94.11
Cu1-O13	2.6472(3)	O13-Cu1-Cl2	104.53
Cu1-N1	1.9754(2)	N1-Cu1-O12	88.06
		O12-Cu1-O13	134.79
		O12-Cu1-Cl1	130.17
		O13-Cu1-N1	54.71
		O13-Cu1-Cl1	84.85
		N1-Cu1-Cl1	99.10

**Figure 4:**
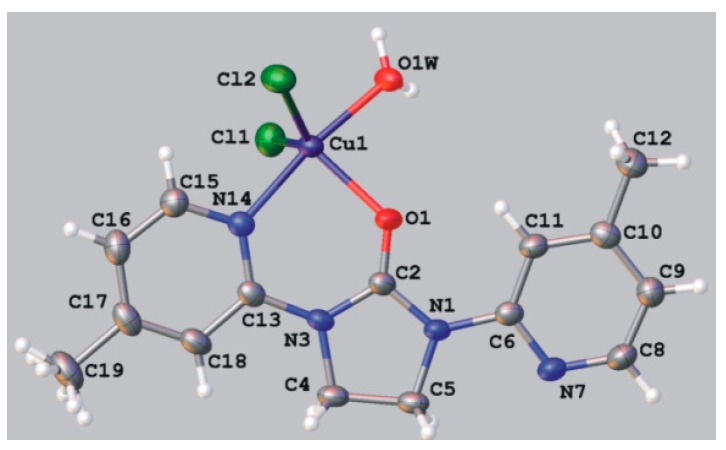
ORTEP representation of molecular structure of **7**.

Interesting five-coordinated mononuclar complex **7** was prepared by reacting equimolar amount of 1,3-*bis*(4-methyl-2-pyridyl)imidazolidin-2-one (**2b**) with copper(II) chloride in methanol containing 1% of water. Elemental analysis data suggested the presence of water molecule in the complex compound, which was confirmed by IR spectrum revealing a broad absorption band with a maximum at 3372 cm^−1^. X-ray analysis (Figure 4, Table 4) indicate that the molecule **7** is not isostructural with **6a**, but it does have a similar molecular structure. Thus, central atom chelated by neutral ligand **2b** and bound to two chloride ions and oxygen of H_2_O. The coordination geometry around the central atom is best described as distorted trigonal bipyramid due to differences in the five Cu-donor bond lengths.

**Table 4:** Selected bond lengths and bond angles in copper complex **7**.

Bond Lengths (Å)		Bond Angles (°)	
Cu1-Cl1	2.362(2)	N14-Cu1-O1	87.58(7)
Cu1-Cl2	2.289(8)	N14-Cu1-Cl1	89.40(6)
Cu1-N14	2.007(8)	N14-Cu1-Cl2	95.83(6)
Cu1-O1	2.042(3)	O1W-Cu1-Cl1	92.45(5)
Cu1-O1W	1.974(4)	O1W-Cu1-O1	84.87(7)
		Cl2-Cu1-O1	119.07(5)
		O1W-Cu1-N14	172.37(8)
		Cl1-Cu1-O1	111.82(4)
		Cl1-Cu1-Cl2	128.99(3)

**Figure 5:**
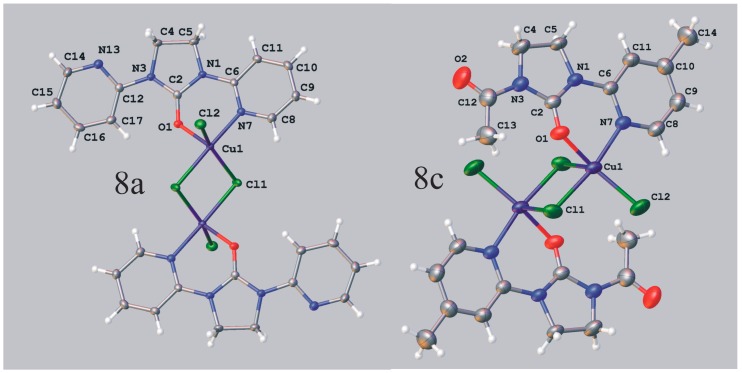
ORTEP representation of molecular structure of **8a** and **8c**. Displacement ellipsoids are shown at the 50% probability level. Only the symmetry independent part of the molecule is labelled.

1,3-*Bis*(2-pyridyl)imidazolidin-2-one (**2b**) and 1-acyl-3-(2-pyridyl)imidazolidin-2-ones (**3a**–**j**) subjected to the reaction with copper(II) chloride gave rise to the formation of the products **8a**–**k**, which appear to be binuclear five-coordinate di-μ-chloro copper(II) complexes. The determination of the three-dimensional structure of the complexes **8a** and **8c** by X-ray diffraction (Figure 5, Table 5) indicates that symmetrical coordination polyhedra of these complexes are square-pyramidal with the central atom displaced from the plane of the four basal atoms towards the apical position. It is noteworthy, however, that the arrangement of ligands in both complexes is different. Thus, in **8c** the apical position is occupied by non-bridging Cl atom which is basal to the other copper in the dimer, while complex **8a** incorporates O atom in that position.

**Table 5:** Selected bond lengths and bond angles in copper complexes **8a** and **8c**.

No.	Bond Lengths (Å)		Bond Angles (°)	
**8a**	Cl1-Cu1	2.3260(5)	Cl1-Cu1-Cl1	85.06(2)
	Cl1-Cu1	2.3064(6)	Cl1-Cu1-Cl2	92.25(2)
	Cl2-Cu1	2.2492(5)	N7-Cu1-Cl2	91.17(5)
	Cu1-N7	2.014(2)	Cl1-Cu1-N7	91.72(5)
	Cu1-O1	2.143(1)	O1-Cu1-Cl1	92.84(4)
			O1-Cu1-Cl1	102.79(4)
			O1-Cu1-N7	86.65(6)
			O1-Cu1-Cl2	106.24(4)
			N7-Cu1-Cl1	176.55(4)
			Cl1-Cu1-Cl2	150.94(2)
**8c**	Cl1-Cu1	2.2889(1)	Cl1-Cu1-O1	85.15
	Cl1-Cu1	2.6261(5)	O1-Cu1-N7	86.80
	Cl2-Cu1	2.2349(1)	Cl2-Cu1-N7	97.38
	Cu1-N7	2.0651(1)	Cl1-Cu1-Cl2	92.09
	Cu1-O1	1.9829(1)	N7-Cu1-Cl1	170.49
			O1-Cu1-Cl2	152.31
			Cl1-Cu1-O1	94.23(1)
			Cl1-Cu1-N7	85.37(1)
			Cl1-Cu1-Cl2	113.35(1)
			Cl1-Cu1-Cl1	90.15(1)

**Figure 6:**
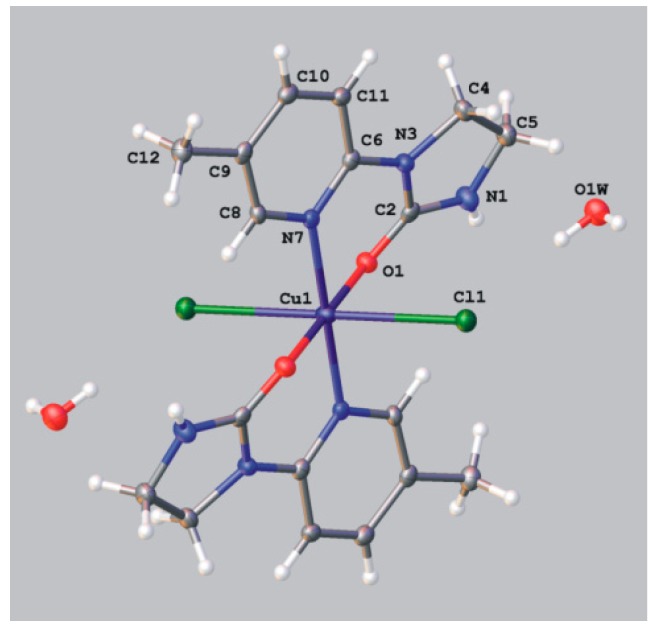
ORTEP representation of molecular structure of **9**. Displacement ellipsoids are shown at the 50% probability level. Only symmetry independent part is labelled.

*N*-(5-methyl-2-pyridyl)imidazolidin-2-one (**1c**) reacted with copper(II) chloride with the formation of mononuclear complex *trans*-CuCl_2_L_2_ (compound **9**, Figure 6, Table 6). A similar structure was previously obtained using *N*,*N*'bis(2-pyridyl)urea [25]. The six-coordinate copper ion sits upon crystallographic inversion center, with ligand chelated through its oxygen atom and 2-pyridyl nitrogen atom. The axial positions are occupied by two chloride ligands. The bond angles around the copper ion are close to 90°, indicating a slight distortion of the octahedral coordination sphere.

**Table 6:** Selected bond lengths and bond angles in copper(II) complex **9**.

Bond Lengths (Å)		Bond Angles (°)	
Cu1-Cl1	2.8254(8)	O1-Cu1-N7	88.31(8)
Cu1-N7	2.019(2)	N7-Cu1-O1	91.69(8)
Cu1-O1	1.950(2)	Cl1-Cu1-O1	92.31(6)
		O1-Cu1-Cl1	87.69(6)
		N7-Cu1-Cl1	90.71(6)
		O1-Cu1-O1	180.00(8)
		N7-Cu1-N7	180.00(9)
		Cl1-Cu1-Cl1	180.00(2)

**Figure 7:**
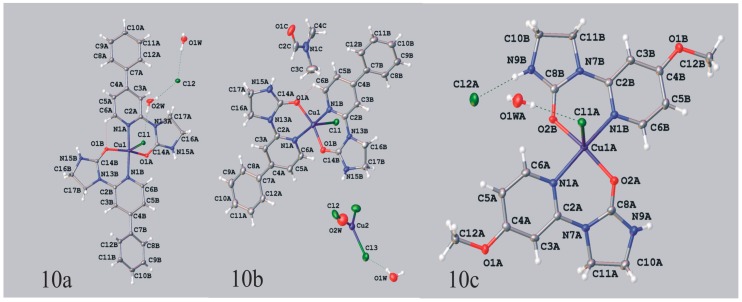
ORTEP representation of molecular structure of **10a** (Cl^−^ counterion), **10b** (CuCl_3_^−^ counterion) and **10c** (Cl^−^ counterion). Hydrogen bonds are shown with dashed lines.

As shown in Table 6 in octahedral complex Cu(II)L_2_Cl_2_ (**9**) the Cu(II)-Cl bonds are elongated (2.8254 Å), and therefore, are susceptible to dissociation. Indeed, in polar solvents ligands **1e** (1-(4-phenyl-2-pyridyl)imidazolidin-2-one) and **1g** (1-(4-methoxy-2-pyridyl)imidazolidin-2-one) subjected to the reaction with copper(II) chloride gave the monocationic complexes **10a/b** and **10c** of general structure [CuL_2_Cl]^+^ whose charge is neutralized by either the Cl^−^ (**10a** and **10c**) or CuCl_3_^−^ (**10b**) ion. Their crystal structures show (Figure 7, Table 7) that two bidentate ligands arranged in *trans* fashion are coordinated to the copper(II) ion through N_(pyridine)_ and O_(imidazolidin-2-one)_ atoms. The fifth coordination comes from chloride ion, and the Cu-Cl bond lengths of 2.4910–2.4877 Å are shorter than those in the octahedral complex **9** discussed above. The geometries around the central atom are best described by square-pyramidal with trigonal-bipyramidal distortion. The counterions,* i.e.*, Cl^−^ in complexes **10a** and **10c** and CuCl_3_^−^ in complex **10b**, remain uncoordinated, however were found to engage in several weak hydrogen-bonding interactions.

**Table 7:** Selected bond lengths and bond angles in copper(II) complexes **10a**, **10b** and **10c**.

No.	Bond Lengths (Å)		Bond Angles (°)	
**10a**	Cu1-Cl1	2.4274(5)	Cl1-Cu1-O1A	101.91(4)
	Cu1-N1A	2.053(2)	Cl1-Cu1-O1B	107.23(4)
	Cu1-N1B	2.041(2)	O1A-Cu1-O1B	150.84(6)
	Cu1-O1A	1.967(1)	N1A-Cu1-O1A	87.52(6)
	Cu1-O1B	1.922(1)	N1A-Cu1-Cl1	94.08(5)
			N1A-Cu1-N1B	171.28(6)
			N1A-Cu1-O1B	89.42(6)
			N1B-Cu1-Cl1	94.58(5)
			N1B-Cu1-O1A	89.74(6)
			N1B-Cu1-O1B	88.95(6)
**10b**	Cu1-Cl1	2.4877(6)	Cl1-Cu1-O1A	111.06(5)
	Cu1-N1A	2.019(2)	Cl1-Cu1-O1B	92.85(5)
	Cu1-N1B	2.015(2)	O1A-Cu1-O1B	156.09(7)
	Cu1-O1A	1.946(2)	N1A-Cu1-O1A	86.87(7)
	Cu1-O1B	1.964(2)	N1A-Cu1-Cl1	97.44(6)
	Cu2-Cl2	2.195(1)	N1A-Cu1-N1B	172.10(8)
	Cu2-Cl3	2.3018(8)	N1A-Cu1-O1B	89.79(7)
			N1B-Cu1-Cl1	90.45(6)
			N1B-Cu1-O1A	89.95(7)
			N1B-Cu1-O1B	90.19(7)
			Cl2-Cu2-Cl3	120.59(4)
			Cl3-Cu2-Cl3	118.81(3)
**10c**	Cu1A-Cl1A	2.4910(5)	N1B-Cu1A-Cl1A	90.04(5)
	Cu1A-N1A	2.031(1)	O2A-Cu1A-Cl1A	105.98(5)
	Cu1A-N1B	2.044(2)	N1A-Cu1A-Cl1A	97.78(5)
	Cu1A-O2A	1.928(2)	O2B-Cu1A-Cl1A	94.12(5)
	Cu1A-O2B	1.948(2)	O2B-Cu1A-N1A	88.03(7)
			N1A-Cu1A-O2A	89.02(7)
			O2A-Cu1A-N1B	89.78(7)
			O2B-Cu1A-N1B	88.65(7)
			O2-Cu1A-O2B	159.90(6)
			N1B-Cu1A-N1A	166.96(7)

### 2.3. In Vitro Antitumor Activity

The* in vitro* antitumor potential of the free ligands **1**–**4** and copper(II) complexes **5**–**10** against human lung cancer (either LCLC-103H or A-427), human bladder cancer (either 5637 or RT-4), human cervical cancer (SISO), and human esophagus cancer (KYSE-520) cell lines was evaluated using a crystal violet microtiter plate assay as described earlier [26]. Primary screening of the new compounds was performed to indicate whether a substance possesses enough activity to inhibit cell growth by 50% at a concentration attainable in cancer cells,* i.e.*, 20 µM.

The free ligands **1**–**4** were inactive, while the complexes of type **5** and **6** obtained from imidazolidin-2-ones, including these substituted with acyl group at the nitrogen atom, showed a remarkable inhibitory activity against lung cancer A-427 cell line (Table 8). It should be pointed out, however, that some copper(II) complexes, although fairly soluble in aprotic polar solvents such as DMF or DMSO, showed rather poor solubility in water and precipitated out of culture media. Therefore, Table 8 incorporates the results of primary screening obtained for the complexes that remained in solution at the test concentration of 20 μM.

**Table 8:** Percent of cell growth relative to untreated control at a concentration of 20 µM (values are averages of three independent determinations with standard deviations, otherwise averages of two determinations without SD. Values were calculated according to Equation (2)).

	Cell Line	LCLC-103H	5637	A-427	SISO	KYSE-520	RT-4
No.							
**5a**		38.74	nd ^†^	35.57	96.68	nd	nd
**5b**		nd	101.05	nd	113.65	95.75	nd
**5e**		88.2 ± 17.4	135.9 ± 43.6	34.9 ± 9.8	nd	nd	nd
**5f**		91.78	nd	79.82	85.30	nd	nd
**5g**		63.17	96.67	44.69	nd	nd	nd
**5h**		36.52	nd	45.11	nd	nd	31.67
**5i**		89.38	33.48	nd	nd	nd	71.86
**5j**		38.28	nd	29.18	nd	nd	41.65
**6a**		101.2 ± 13.1	106.2 ± 31.1	48.30 ± 11.13	nd	nd	nd
**6b**		91.5 ± 8.8	87.4 ± 20.6	−40.6 ± 31.8	nd	nd	nd
**6c**		75.9 ± 21.7	104.05 ± 40.54	30.4 ± 10.1	nd	nd	nd
**6d**		78.4 ± 21.2	145.6 ± 30.8	26.6 ± 11.3	nd	nd	nd
**7**		66.93	93.78	nd	nd	nd	94.21
**8a**		27.45	nd	18.30	89.69	nd	nd
**9**		69.23 ± 23.21	135.88 ± 10.66	75.12 ± 13.31	nd	nd	nd
**10c**		216.63 ± 14.43	105.96 ± 24.06	79.4 ± 6.26	nd	nd	nd

Note: **^†^** nd: = not determined.

For secondary screening aimed at determining cytotoxic potencies (IC_50_) we selected imidazolidine-2-thione complexes **5h** and **5j** which exhibited a pronounced activity against at least three cancer cell lines. The results of secondary screenings are presented in Table 9. Thus, for complexes **5h** and **5j**, both of which exhibited growth inhibitory effects against LCLC-103H, A-427, SISO, RT-4 and DAN-G cell lines, the calculated IC_50_ values were in the range of 8–25 µM. It is worth noting that most active (IC_50_ in the range of 8.55–12.80 µM) was compound **5j** containing *tert*-butyl substituent at the position 4 of pyridine ring. This observation is in line with recent findings that an electron-donating *tert*-butyl group may stabilize a copper(II) complexes by increasing the electron density at the central ion which, in turn, elicits a “self-activating” mechanism of DNA strand scission through the generation of reactive oxygen species (ROS) that are possibly responsible for the DNA cleavage [27,28,29]. Further work will be needed to confirm this, however.

**Table 9:** IC_50_ (µM) values in five human cancer cell lines obtained after 96 h exposure **^†^**.

	Cell Line	LCLC-103H	A-427	SISO	RT-4	DAN-G
No.						
**5h**		11.16 ± 3.20	24.38 ± 14.28	24.81 ± 13.78	8.25 ± 3.79	24.88 ± 3.04
**5j**		11.71 ± 5.06	8.55 ± 3.10	10.83 ± 3.05	9.64 ± 4.95	12.80 ± 0.64
*cisplatin* *		0.90 ± 0.19	1.96 ± 0.54	0.24 ± 0.06	1.61 ± 0.16	0.73 ± 0.34

Notes: **^†^** Values are average of three independent determinations with standard deviations; * Ref. [26].

## 3. Experimental Section

Melting points both the ligands and copper(II) complexes were determined on a Boetius apparatus and are uncorrected. FT-IR spectra were measured by Nicolet-380 spectrophotometer and ^1^H-NMR and ^13^C-NMR spectra were recorded on a Varian Gemini instrument operating at 200 MHz and 50 MHz, respectively, in CDCl_3_ or DMSO-*d*_6_ as a solvent. Chemical shifts are shown in parts per million (ppm) on the δ scale. Coupling constants are shown in hertz (Hz).

Chromatographic separations were performed on silica gel 60 PF_254_ containing gypsum (Merck) by use of chromatotron or flash column chromatography (silica gel 0.040–0.063 mm). Thin-layer chromatography (TLC) was performed with Merck silica gel plates and spots were visualized with UV light at 254 nm.

The diffraction data for single crystals were collected with KM4CCD, Oxford Diffraction Xcalibur or Oxford Diffraction SuperNova diffractometers. The intensity data were processed using the CrysAlis software [30]. The structures were solved by direct methods with the program SHELXS-97 [31] and refined by full-matrix least-squares method on *F*^2^ with SHELXL-97 [31].

Crystallographic data for compounds have been deposited with the Cambridge Crystallographic Data Centre, with the deposition Nos CCDC 986094, 986095, 986193–986202. These data can be obtained free of charge from The Cambridge Crystallographic Data Centre.

Elemental analyses of C, H and N were within ±0.4% of the theoretical values.

All cell culture reagents were purchased from Sigma (Deisenhofen, FRG). Cancer cell lines: human large cell lung carcinoma LCLC-103H, human urinary bladder carcinoma 5637, human lung carcinoma A-427, human uterine cervical adenocarcinoma SISO, esophageal squamous cell carcinoma KYSE-520, human bladder cell carcinoma RT-4 and human pancreas cell adenocarcinoma DAN-G were obtained from the German Collection of Microorganisms and Cell Cultures (DSMZ, Brauschweig, FRG). The culture medium for cell lines was RPMI-1640 medium containing 2 g/L HCO_3_, and 10% FCS. Cells were grown in 75 cm^2^ plastic culture flasks (Sarstedt, Nümbrecht, FRG) in a humid atmosphere of 5% CO_2_ at 37 °C and were passaged shortly before becoming confluent.

For the cytotoxicity studies, 100 μL of a cell suspension were seeded into 96-well microtiter plates (Sarstedt) at a density of 1000 cell per well except for the LCLC-103H cell line, which was plated out at 250 cells per well. One day after plating, the cells were treated with test substance at five concentrations per compound. The 1000-fold concentrated stock solutions in DMF or DMSO were serially diluted by 50% in DMF or DMSO to give the feed solutions, which were diluted 500-fold into culture medium. The controls received just DMF or DMSO. Each concentration was tested in eight wells, with each well receiving 100 μL of the medium containing the substance. The concentration ranges were chosen to bracket the expected IC_50_ values as best as possible. Cells were then incubated for 96 h, after which time the medium was removed and replaced with 1% glutaraldehyde/PBS. Staining with crystal violet was done as previously described [26]. O.D. was measured at λ = 570 nm with an Anthos 2010 plate reader (Salzburg, Austria).

Corrected T/C values were calculated using MS Excel 2007 program by the equation:

(*T/C*)_corr_(%) = (O.D._T_ − O.D._c.0_)/(O.D._C_ − O.D._c.0_) × 100 (2)

where O.D._T_ is the mean absorbance of the treated cells; O.D._C_ the mean absorbance of the controls and O.D._c.0_ the mean absorbance at the time drug was added. The IC_50_ values were estimated by a linear least-square regression of the *T/C*_corr_ values* versus* the logarithm of the substance concentration; only concentrations that yielded *T/C*_corr_ values between 10% and 90% were used in the calculation. The reported IC_50_ values are the averages of 3 independent experiments.

### 3.1. Synthesis of 1-Acyl-3-(2-pyridyl)imidazolidin-2-ones ***3a**–**j*** (General Procedure)

Imidazolidin-2-one (0.001 mol) was refluxed in 5 mL of acetic anhydride or butyric anhydride for 6 h. The reaction mixture was concentrated under reduced pressure and basified with 20% solution of K_2_CO_3_. Precipitated was collected by suction, washed with water and dried. In case when oily residue was formed after addition of K_2_CO_3_ the product was extracted with chloroform (3 × 15 mL), dried with anhydrous MgSO_4_, filtrated and concentrated under reduced pressure. Product was purified by use of chromatotron, flash column chromatography or crystallization. According to described general procedure were obtained following compounds:

*1-Acetyl-3-(5-methyl-2-pyridyl)imidazolidin-2-one* (**3a**). Compound **3a** was purified by use of chromatotron (eluent: dichloromethane/ethyl acetate, 7:3, v/v); yield 60%; mp. 179–181 °C; IR (KBr) ν [cm^−1^]: 2999, 2953, 2922, 2853, 1731, 1680, 1484, 1403, 1375, 1291, 1246, 1023; ^1^H-NMR (500 MHz, CDCl_3_): δ 2.30 (s, 3H, CH_3_), 2.57 (s, 3H, CH_3_), 3.93 (t, 2H, CH_2_), 4.10 (t, 2H, CH_2_), 7.54 (d, *J* = 8.3 Hz, 1H, Ar-H), 8.13 (d, *J* = 8.3 Hz, 1H, Ar-H), 8.16 (s, 1H, Ar-H); Anal. Calcd. for C_11_H_13_N_3_O_2_: C, 60.26; H, 5.98; N, 19.17; Found: C, 60.11; H, 5.78; N, 19.08.

*1-Acetyl-3-(4-methyl-2-pyridyl)imidazolidin-2-one* (**3b**). Compound **3b** was purified by use of chromatotron (eluent: dichloromethane/acetone, 95:5, v/v); yield 70%; mp. 152–154 °C; IR (KBr) ν [cm^−1^]: 3017, 2920, 1732, 1679, 1605, 1485, 1426, 1373, 1296, 1249, 1195; ^1^H-NMR (200 MHz, (CD_3_)_2_SO): δ 2.33 (s, 3H, CH_3_), 2.43 (s, 3H, OCH_3_), 3.76 (t, 2H, CH_2_), 3.96 (t, 2H, CH_2_), 6.97 (d, *J* = 4.5 Hz, 1H, Ar-H), 8.02 (s, 1H, Ar-H), 8.22 (d, *J* = 4.5 Hz, 1H, Ar-H); Anal. Calcd. for C_11_H_13_N_3_O_2_: C, 60.26; H, 5.98; N, 19.17; Found: C, 60.08; H, 5.81; N, 18.96.

*1-Butyryl-3-(4-methyl-2-pyridyl)imidazolidin-2-one* (**3c**). Compound **3c** was purified by use of chromatotron (eluent: chloroform); yield 54%; mp. 82–83 °C; IR (KBr) ν [cm^−1^]: 3058, 2967, 2915, 2878, 1734, 1677, 1603, 1560, 1374, 1323, 1246, 1190; ^1^H-NMR (500 MHz, CDCl_3_): δ 1.00 (t, 3H, CH_3_), 1.73 (sextet, 2H, CH_2_), 2.38 (s, 3H, CH_3_), 2.97 (t, 2H, CH_2_), 3.93 (t, 2H, CH_2_), 4.10 (t, 2H, CH_2_), 6.87 (d, *J* = 5.4 Hz, 1H, Ar-H), 8.08 (s, 1H, Ar-H), 8.19 (d, *J* = 5.4 Hz, 1H, Ar-H); Anal. Calcd. for C_13_H_17_N_3_O_2_: C, 63.14; H, 6.93; N, 16.99; Found: C, 62.99; H, 6.81; N, 16.89.

*1-Acetyl-3-(4-tert-butyl-2-pyridyl)imidazolidin-2-one* (**3d**). Compound **3d** was purified by use of chromatotron (eluent: dichloromethane/ethyl acetate, 4:1, v/v); yield 59%; mp. 163–166 °C; IR (KBr) ν [cm^−1^]: 2970, 2919, 2870, 1726, 1685, 1598, 1547, 1484, 1420, 1374, 1293, 1252, 1120; ^1^H-NMR (200 MHz, CDCl_3_): δ 1.35 (s, 9H, C(CH_3_)_3_), 2.60 (s, 3H, CH_3_), 3.94–3.98 (m, 2H, CH_2_), 4.10–4.14 (m, 2H, CH_2_), 7.08 (dd, *J*_1_ = 1.3 Hz, *J*_2_ = 5.4 Hz, 1H, Ar-H), 8.26 (d, *J* = 5.4 Hz, 1H, Ar-H), 8.30 (s, 1H, Ar-H); Anal. Calcd. for C_14_H_19_N_3_O_2_: C, 64.35; H, 7.33; N, 16.08; Found: C, 64.17; H, 7.19; N, 15.86.

*1-Acetyl-3-(4-phenyl-2-pyridyl)imidazolidin-2-one* (**3e**). Compound **3e** was purified by use of flash column chromatography (eluent: chloroform/ethyl acetate:methanol, 5:2:1, v/v/v); yield 76%; mp. 156–157 °C; IR (KBr) ν [cm^−1^]: 3067, 3021, 2960, 2918, 1747, 1685, 1594, 1545, 1474, 1420, 1377, 1368, 1308, 1251; ^1^H-NMR (200 MHz, (CD_3_)_2_SO): δ 2.45 (s, 3H, CH_3_), 3.77–3.85 (m, 2H, CH_2_), 3.99–4.07 (m, 2H, CH_2_), 7.45–7.58 (m, 4H, Ar-H), 7.72–7.77 (m, 2H, Ar-H), 8.43–8.48 (m, 2H, Ar-H), ^13^C-NMR (50 MHz, (CD_3_)_2_SO): δ 23.98, 38.55, 41.05, 110.29, 117.28, 127.07 (two overlapping signals), 129.58 (two overlapping signals), 129.66, 137.72, 148.68, 149.18, 152.32, 153.02, 170.09; Anal. Calcd. for C_16_H_15_N_3_O_2_: C, 68.31; H, 5.37; N, 14.94; Found: C, 68.22; H, 5.23; N, 14.78.

*1-Butyryl-3-(4-phenyl-2-pyridyl)imidazolidin-2-one* (**3f**). Compound **3f** was purified by use of chromatotron (eluent: chloroform); yield 70%; mp. 155–156 °C; IR (KBr) ν [cm^−1^]: 3110, 3069, 2962, 2918, 2872, 1735, 1682, 1593, 1474, 1374, 1248, 1224; ^1^H-NMR (500 MHz, CDCl_3_): δ 1.02 (t, 3H, CH_3_), 1.75 (sextet, 2H, CH_2_), 3.00 (t, 2H, CH_2_), 3.97 (t, 2H, CH_2_), 4.16 (t, 2H, CH_2_), 7.28 (d, *J* = 5.4 Hz, 1H, Ar-H), 7.43–7.50 (m, 3H, Ar-H), 7.70 (d, *J* = 7.8 Hz, 2H, Ar-H), 8.39 (d, *J* = 5.4 Hz, 1H, Ar-H), 8.53 (s, 1H, Ar-H); Anal. Calcd. for C_18_H_19_N_3_O_2_: C, 69.88; H, 6.19; N, 13.58; Found: C, 69.79; H, 6.02; N, 13.50.

*1-Acetyl-3-[4-(3-phenylpropyl)-2-pyridyl]imidazolidin-2-one* (**3g**). Compound **3g** was purified by use of chromatotron (eluent: dichloromethane/ethyl acetate, 4:1, v/v); yield 64%; mp. 89–90 °C; IR (KBr) ν [cm^−1^]: 3060, 3024, 2943, 2925, 2858, 1722, 1683, 1601, 1560, 1483, 1438, 1402, 1379, 1305, 1278, 1254; ^1^H-NMR (500 MHz, (CD_3_)_2_SO): δ 1.90 (q, 2H, CH_2_), 2.45 (s, 3H, CH_3_), 2.60–2.64 (m, 4H, 2×CH_2_), 3.79 (t, 2H, CH_2_), 3.98 (t, 2H, CH_2_), 7.02 (d, *J* = 4.9 Hz, 1H, Ar-H), 7.16–7.29 (m, 5H, Ar-H), 8.07 (s, 1H, Ar-H), 8.27 (d, *J* = 4.9 Hz, 1H, Ar-H); Anal. Calcd. for C_19_H_21_N_3_O_2_: C, 70.57; H, 6.55; N, 12.99; Found: C, 70.43; H, 6.51; N, 13.20. 

*1-Acetyl-3-(4-methoxy-2-pyridyl)imidazolidin-2-one* (**3h**). Compound **3h** was purified by use of chromatotron (eluent: dichloromethane/ethyl acetate, 4:1, v/v); yield 87%; mp. 139–140 °C; IR (KBr) ν [cm^−1^]: 3023, 2983, 2920, 1731, 1687, 1594, 1566, 1455, 1406, 1382, 1309, 1257, 1223, 1179; ^1^H-NMR (500 MHz, (CD_3_)_2_SO): δ 2.44 (s, 3H, CH_3_), 3.78 (t, 2H, CH_2_), 3.84 (s, 3H, OCH_3_), 3.97 (t, 2H, CH_2_), 6.77 (dd, *J*_1_ = 1.9 Hz, *J*_2_ = 5.9 Hz, 1H, Ar-H), 7.77 (d, *J* = 1.9 Hz, 1H, Ar-H), 8.19 (d, *J* = 5.9 Hz, 1H, Ar-H); Anal. Calcd. for C_11_H_13_N_3_O_3_: C, 56.16; H, 5.57; N, 17.86; Found: C, 55.99; H, 5.51; N, 17.80.

*1-Butyryl-3-(4-methoxy-2-pyridyl)imidazolidin-2-one* (**3i**). Compound **3i** was purified by use of chromatotron (eluent: chloroform); yield 66%; mp. 112–113 °C; IR (KBr) ν [cm^−1^]: 3112, 3016, 2964, 2921, 2875, 1736, 1691, 1595, 1564, 1482, 1398, 1375, 1255, 1216, 1178; ^1^H-NMR (200 MHz, CDCl_3_): δ 1.01 (t, 3H, CH_3_), 1.72 (sextet, 2H, CH_2_), 2.98 (t, 2H, CH_2_), 3.89 (s, 3H, OCH_3_), 3.92–4.00 (m, 2H, CH_2_), 4.07–4.16 (m, 2H, CH_2_), 6.61 (dd, *J*_1_ = 2.2 Hz, *J*_2_ = 5.9 Hz, 1H, Ar-H), 7.86 (d, *J* = 2.2 Hz, 1H, Ar-H), 8.14 (d, *J* = 5.9 Hz, 1H, Ar-H); Anal. Calcd. for C_13_H_17_N_3_O_3_: C, 59.30; H, 6.51; N, 15.96; Found: C, 59.21; H, 6.46; N, 16.00.

*1-Acetyl-3-(4-benzyloxy-2-pyridyl)imidazolidin-2-one* (**3j**). Compound **3j** was purified by use of chromatotron (eluent: chloroform/ethyl acetate, 4:1, v/v); yield 70%; mp. 151–153 °C; IR (KBr) ν [cm^−1^]: 3101, 3069, 3027, 2916, 1743, 1737, 1673, 1596, 1563, 1481, 1452, 1386, 1318, 1255, 1215, 1011, 868; ^1^H-NMR (500 MHz, (CD_3_)_2_SO): δ 2.45 (s, 3H, OCH_3_), 3.78 (t, 2H, CH_2_), 3.98 (t, 2H, CH_2_), 5.21 (s, 2H, OCH_2_), 6.86 (dd, *J*_1_ = 1.9 Hz, *J*_2_ = 5.9 Hz, 1H, Ar-H), 7.36–7.49 (m, 5H, Ar-H), 7.87 (d, *J* = 1.9 Hz, 1H, Ar-H), 8.21 (d, *J* = 5.9 Hz, 1H, Ar-H); Anal. Calcd. for C_17_H_17_N_3_O_3_: C, 65.58; H, 5.50; N, 13.50; Found: C, 65.42; H, 5.28; N, 13.71.

### 3.2. Synthesis of N-(2-Pyridyl)imidazolidine-2-thiones ***4a**–**c***, ***e**–**g*** (General Procedure)

Appropriate* N*-(2-pyridyl)imidazolidin-2-one (0.001 mol) was refluxed with Lawesson’s reagent (0.00075 mol) in anhydrous toluene (8 mL) for 12 h and concentrated under reduced pressure. The residue was extracted with chloroform (2 × 20 mL), dried with anhydrous MgSO_4_, filtrated and concentrated under reduced pressure. Product was separated from oily residue by use of chromatotron. According above given procedure were obtained following compounds:

*1-(2-Pyridyl)imidazolidine-2-thione* (**4a**). Compound **4a** was purified by use of chromatotron (eluent: chloroform/ethyl acetate/acetone, 8:1:1, v/v/v); yield 50%; mp. 99–102 °C; IR (KBr) ν [cm^−1^]: 3196, 3036, 2993, 1591, 1567, 1533, 1466, 1438, 1413, 1347, 1228; ^1^H-NMR (200 MHz, CDCl_3_): δ 3.68 (t, 2H, CH_2_); 4.44 (t, 2H, CH_2_); 6.96 (bs, 1H, NH); 7.01–7.07 (m, 1H, Ar-H); 7.64–7.73 (m, 1H, Ar-H); 8.33–8.36 (m, 1H, Ar-H); 8.93 (d, *J* = 8.0 Hz, 1H, Ar-H); ^13^C-NMR (50 MHz, CDCl_3_): δ 40.93, 49.43, 116.33, 119.62, 136.69, 147.32, 152.32, 181.27; Anal. Calcd. for C_8_H_9_N_3_S: C, 53.61; H, 5.06; N, 23.44; Found: C, 53.54; H, 4.92; N, 23.37.

*1-(6-Methyl-2-pyridyl)imidazolidine-2-thione* (**4b**). Compound **4b** was purified by use of chromatotron (eluent: dichloromethane/ethyl acetate, 1:1, v/v); yield 31%; mp. 133–135 °C; IR (KBr) ν [cm^−1^]: 3222, 3101, 3029, 2974, 1588, 1519, 1456, 1428, 1397, 1346, 1254, 1231; ^1^H-NMR (200 MHz, (CD_3_)_2_SO): δ 2.41 (s, 3H, CH_3_), 3.53 (t, 2H, CH_2_), 4.26 (t, 2H, CH_2_), 6.97 (d, *J* = 7.3 Hz, 1H, Ar-H), 7.64 (t, 1H, Ar-H), 8.60 (d, *J* = 8.4 Hz, 1H, Ar-H), 9.00 (s, 1H, NH); ^13^C-NMR (50 MHz, (CD_3_)_2_SO): δ 24.25, 41.05, 49.09, 113.18, 118.54, 137.04, 152.14, 156.09, 180.19; Anal. Calcd. for C_9_H_11_N_3_S: C, 55.93; H, 5.74; N, 21.74; Found: C, 55.86; H, 5.64; N, 21.48.

*1-(5-Methyl-2-pyridyl)imidazolidine-2-thione* (**4c**). Compound **4c** was purified by use of chromatotron (eluent: dichloromethane/ethyl acetate, 7:3, v/v); yield 51%; mp. 200–203 °C; IR (KBr) ν [cm^−1^]: 3271, 2969, 2904, 1608, 1570, 1514, 1479, 1388, 1341, 1239, 1217; ^1^H-NMR (200 MHz, (CD_3_)_2_SO): δ 2.26 (s, 3H, CH_3_); 3.53 (t, 2H, CH_2_); 4.24 (t, 2H, CH_2_); 7.59 (dd, *J*_1_ = 2.1 Hz, *J*_2_ = 8.5 Hz, 1H, Ar-H), 8.18 (s, 1H, Ar-H), 8.67 (d, *J* = 8.5 Hz, 1H, Ar-H), 8.96 (s, 1H, NH); ^13^C-NMR (50 MHz, (CD_3_)_2_SO): δ 17.52, 40.65, 49.12, 115.84, 128.49, 137.28, 147.28, 150.70, 180.15; Anal. Calcd. for C_9_H_11_N_3_S: C, 55.93; H, 5.74; N, 21.74; Found: C, 55.81; H, 5.63; N, 21.68.

*1-(4-Tert-butyl-2-pyridyl)imidazolidine-2-thione* (**4e**). Compound **4e** was purified by use of chromatotron (eluent: dichloromethane/ethyl acetate, 7:3, v/v); yield 50%; mp. 160–163 °C; IR (KBr) ν [cm^−1^]: 3197, 3018, 2962, 2927, 2859, 1602, 1548, 1521, 1482, 1412, 1311, 1236, 1119, 830, 553; ^1^H-NMR (200 MHz, CDCl_3_): δ 1.34 (s, 9H, 3×CH_3_), 3.68 (t, 2H, CH_2_), 4.46 (t, 2H, CH_2_), 6.76 (br.s, 1H, NH), 7.06 (dd, *J*_1_ = 1.6 Hz, *J*_2_ = 5.5 Hz, 1H, Ar-H), 8.25 (d, *J* = 5.5 Hz, 1H, Ar-H), 9.01 (d, *J* = 1.6 Hz, 1H, Ar-H); ^13^C-NMR (50 MHz, CDCl_3_): δ 31.02 (three overlapping signals), 35.73, 41.52, 50.06, 114.33, 117.67, 147.21, 152.85, 161.89, 181.86; Anal. Calcd. for C_12_H_17_N_3_S: C, 61.24; H, 7.28; N, 17.85; Found C, 61.02; H, 6.18; N, 17.78.

*1-(4-Phenyl-2-pyridyl)imidazolidine-2-thione* (**4f**). Compound **4f** was purified by use of chromatotron (eluent: dichloromethane/ethyl acetate, 4:1, v/v); yield 51%; mp. 201–202 °C; IR (KBr) ν [cm^−1^]: 3204, 3026, 2969, 2924, 1597, 1532, 1466, 1412, 1230; ^1^H-NMR (200 MHz, (CD_3_)_2_SO): δ 3.58 (t, 2H, CH_2_), 4.33 (t, 2H, CH_2_), 7.44–7.60 (m, 4H, Ar-H), 7.73–7.77 (m, 2H, Ar-H), 8.43 (d, *J* = 5.2 Hz, 1H, Ar-H), 9.16 (s, 1H, NH), 9.25 (s, 1H, Ar-H); ^13^C-NMR (50 MHz, (CD_3_)_2_SO): δ 41.06, 49.12, 113.36, 117.10, 127.01 (two overlapping signals), 129,58 (three overlapping signals), 137.82, 147.69, 148.29, 153.54, 180.19; Anal. Calcd. for C_14_H_13_N_3_S: C, 65.85; H, 5.13; N, 16.46; Found: C, 65.78; H, 5.04; N, 16.24.

*1-(6-Methoxy-2-pyridyl)imidazolidine-2-thione* (**4g**). Compound **4g** was purified by use of chromatotron (eluent: chloroform); yield 43%; mp. 186–190 °C; IR (KBr) ν [cm^−1^]: 3365, 3008, 2947, 1594, 1584, 1431, 1397, 1361, 1247; ^1^H-NMR (500 MHz, (CD_3_)_2_SO): δ 3.56 (t, 2H, CH_2_), 3.84 (s, 3H, OCH_3_), 4.32 (t, 2H, CH_2_), 6.53 (d, *J* = 7.8 Hz, 1H, Ar-H), 7.68 (t, 1H, Ar-H), 8.47 (d, *J* = 7.8 Hz, 1H, Ar-H), 9.05 (s, 1H, NH); ^13^C-NMR [50 MHz, (CD3)2SO]: δ 39.40, 48.88, 53.20, 104.42, 107.59, 139.81, 150.72, 162.09, 179.98; Anal. Calcd. for C_9_H_11_N_3_OS: C, 51.65; H, 5.30; N, 20.08; Found: C, 51.52; H, 5.24; N, 19.79.

### 3.3. Synthesis of Copper(II) Complexes ***5a**–**8k*** (General Procedure)

To a solution of appropriate ligand in 5 mL of ethanol or methanol was added dropwise at ambient temperature, copper(II) chloride dissolved in 1 mL of ethanol or methanol (in 1:1 molar ratio). The solution was left at room temperature and then the solvent was slowly evaporated. The resulting precipitate (a few minutes to 48 h) was filtered and washed with ethanol or methanol and dried in a desiccator. The following complexes were prepared according to above given procedure:

*Dichloro[1-(2-pyridyl)imidazolidin-2-one]copper(II)* (**5a**). Solvent: ethanol, dark green crystals, yield 55%; mp. 241–245 °C; IR (KBr) ν [cm^−1^]: 3251, 3126, 2923, 1675, 1606, 1474, 1451, 1436, 1317, 1284, 1170, 769; Anal. Calcd. for C_8_H_9_Cl_2_CuN_3_O (297.63): C, 32.28; H, 3.05; N, 14.12; Found: C, 32.14; H, 2.91; N, 13.79.

*Dichloro[1-(6-methyl-2-pyridyl)imidazolidin-2-one]copper(II)* (**5b**). Solvent: methanol, dark brown crystals; mp. 211–215 °C; IR (KBr) ν [cm^−1^]: 3316, 3069, 2920, 1662, 1604, 1494, 1443, 1351, 1285, 1087; Anal. Calcd. for C_9_H_11_Cl_2_CuN_3_O (311.65): C, 34.68; H, 3.56; N, 13.48; Found: C, 34.42; H, 3.50; N, 13.28.

Crystal data for **5b** CCDC no. 986196: C_9_H_11_Cl_2_CuN_3_O, *M* = 311.65, monoclinic, space group *P*2_1_/n (no. 14), *Z* = 4, *a* = 6.7379(2) Å, *b* = 16.9634(3) Å, *c* = 9.9351(2) Å, β = 105.290(2), *V* = 1095.36(4) Å^3^, *T* = 100 K, μ(MoKα) = 2.460 mm^−1^, 12880 reflections measured, 2816 unique (*R*_int_ = 0.0200) which were used in all calculations. The final *wR*_2_ was 0.0630 (all data) and *R*_1_ was 0.0217 [I > 2σ (I)].

*Dichloro[1-(4-methyl-2-pyridyl)imidazolidin-2-one]copper(II)* (**5c**). Solvent: methanol, light green crystals, yield 65%; mp. 237–238 °C; IR (KBr) ν [cm^−1^]: 3202, 1658, 1625, 1508, 1480, 1461, 1317, 1290, 1249, 1024, 828, 818, 742; Anal. Calcd. for C_9_H_11_Cl_2_CuN_3_O (311.66): C, 34.68; H, 3.56; N, 13.48; Found: C, 34.42; H, 3.46; N, 13.17.

*Dichloro[1-(4-methoxy-2-pyridyl)imidazolidin-2-one]copper(II)* (**5d**). Solvent: ethanol, green crystals, yield 47%; mp. 223–227 °C; IR (KBr) ν [cm^−1^]: 3185, 2975, 1678, 1619, 1561, 1485, 1474, 1455, 1294, 1062, 1029, 833, 751, 737; Anal. Calcd. for C_9_H_11_Cl_2_CuN_3_O_2_ (327.65): C, 32.99; H, 3.38; N, 12.82; Found: C, 32.86; H, 3.32; N, 12.48.

*Dichloro[1-(4-ethoxy-6-methyl-2-pyridyl)imidazolidin-2-one]copper(II)* (**5e**). Solvent: ethanol, brown crystals, yield 87%; mp. 189–193 °C; IR (KBr) ν [cm^−1^]: 3336, 2985, 1673, 1612, 1459, 1430, 1300, 1205, 1154, 1047, 851, 837, 744, 715, 636; Anal. Calcd. for C_11_H_15_Cl_2_CuN_3_O_2_ (355.71): C, 37.14; H, 4.25; N, 11.81; Found: C, 37.02; H, 4.17; N, 11.68.

*Dichloro[1-(2-pyridyl)imidazolidine-2-thione]copper(II)* (**5f**). Solvent: ethanol, dark green crystals, yield 74%; mp. 195–198 °C; IR (KBr) ν [cm^−1^]: 3198, 1601, 1575, 1540, 1466, 1440, 1419, 1353, 1323, 1238, 777, 670, 543; Anal. Calcd. for C_8_H_9_Cl_2_CuN_3_S (313.69): C, 30.63; H, 2.89; N, 13.40; Found: C, 30.52; H, 2.86; N, 13.76.

*Dichloro[1-(6-methyl-2-pyridyl)imidazolidine-2-thione]copper(II)* (**5g**). Solvent: ethanol, dark green crystals, yield 65%; mp. 230–233 °C; IR (KBr) ν [cm^−1^]: 3322, 3064, 1662, 1604, 1462, 1444, 1351, 1286, 1087, 801, 747, 734; Anal. Calcd. for C_9_H_11_Cl_2_CuN_3_S (327.72): C, 32.98; H, 3.38; N, 12.82; Found: C, 32.88; H, 3.30; N, 12.58.

*Dichloro[1-(5-methyl-2-pyridyl)imidazolidine-2-thione]copper(II)* (**5h**). Solvent: ethanol, dark green crystals, yield 59%; mp. 186–190 °C; IR (KBr) ν [cm^−1^]: 3202, 3058, 2962, 2912, 1613, 1539, 1504, 1429, 1385, 1321, 1232, 1053, 821; Anal. Calcd. for C_9_H_11_Cl_2_CuN_3_S (327.71): C, 32.98; H, 3.38; N, 12.82; Found: C, 32.84; H, 3.27; N, 12.61.

Crystal data for **5h** CCDC no. 986201: C_9_H_11_Cl_2_CuN_3_S, *M* = 327.71, triclinic, space group *P*-1 (no. 2), *Z* = 2, *a* = 8.1363(3) Å, *b* = 9.0505(4) Å, *c* = 9.9441(3) Å, α = 63.452(4), β = 77.213(3), γ = 69.989(4), *V* = 613.46(4) Å^3^, *T* = 296 K, μ(CuKα) = 7.908 mm^−1^, 12399 reflections measured, 2531 unique (*R*_int_ = 0.0378) which were used in all calculations. The final *wR*_2_ was 0.0942 (all data) and *R*_1_ was 0.0318 [I > 2σ (I)].

*Dichloro[1-(4-methyl-2-pyridyl)imidazolidine-2-thione]copper(II)* (**5i**). Solvent: methanol, dark green crystals; mp. 179–181 °C; IR (KBr) ν [cm^−1^]: 3174, 3070, 1618, 1566, 1547, 1440, 1347, 1239; Anal. Calcd. for C_9_H_11_Cl_2_CuN_3_S (327.71): C, 32.98; H, 3.38; N, 12.82; Found: C, 32.84; H, 3.32; N, 12.78.

Crystal data for **5i** CCDC no. 986193: C_9_H_11_Cl_2_CuN_3_S, *M* = 327.71, monoclinic, space group *P*2_1_/c (no. 14), *Z* = 4, *a* = 8.4124(4) Å, *b* = 13.8054(6) Å, *c* = 11.4288(5) Å, β = 110.782(4), *V* = 1240.94(1) Å^3^, *T* = 140 K, μ(MoKα) = 2.333 mm^−1^, 10224 reflections measured, 2538 unique (*R*_int_ = 0.0189) which were used in all calculations. The final *wR*_2_ was 0.0545 (all data) and *R*_1_ was 0.0201 [I > 2σ (I)].

*Dichloro[1-(4-tert-butyl-2-pyridyl)imidazolidine-2-thione]copper(II)* (**5j**). Solvent: methanol, dark green crystals, yield 76%; mp. 165–168 °C; IR (KBr) ν [cm^−1^]: 3163, 3054, 2957, 2923, 1615, 1554, 1536, 1442, 1294, 1247, 1020, 863, 843; Anal. Calcd. for C_12_H_17_Cl_2_CuN_3_S (369.79): C, 38.97; H, 4.63; N, 11.36; Found: C, 38.92; H, 4.58; N, 11.21.

Crystal data for **5j** CCDC no. 986094: C_12_H_17_Cl_2_CuN_3_S, *M* = 369.79, orthorhombic, space group *P*bca (no. 61), *Z* = 8, *a* = 14.6475(9) Å, *b* = 11.3493(8) Å, *c* = 18.1891(13) Å, *V* = 3023.7(4) Å^3^, *T* = 130 K, μ(CuKα) = 6.490 mm^−1^, 16551 reflections measured, 3117 unique (*R*_int_ = 0.0944) which were used in all calculations. The final *wR*_2_ was 0.1678 (all data) and *R*_1_ was 0.0583 (I > 2σ (I)). In the diffraction pattern reflections with l = 2n + 1 were weak. The structure is strongly disordered with the complex molecule adopting three different overlapping orientations. The main orientation has an occupancy of 0.689(4) and the remaining ones 0.153(4) and 0.158(4). The atoms forming the minor orientation of the molecule were refined with a common isotropic temperature factor, except Cu, Cl and S atoms which were refined anisotropically. The geometry of the molecules in minor orientation was restricted to be the same as for the major orientation. Some restraints were also imposed on the planar fragments of the molecules.

*Dichloro[1-(4-phenyl-2-pyridyl)imidazolidine-2-thione]copper(II)* (**5k**). Solvent: ethanol, dark green, yield 59%; mp. 165–170 °C; IR (KBr) ν [cm^−1^]: 3207, 3052, 3004, 2960, 1613, 1552, 1466, 1437, 1276, 1236, 763, 696, 556; Anal. Calcd. for C_14_H_13_Cl_2_CuN_3_S (389.79): C, 43.14; H, 3.36; N, 10.78; Found: C, 42.99; H, 3.26; N, 10.43.

*Dichloro[1-(6-methoxy-2-pyridyl)imidazolidin-2-one]copper(II)* (**6a**). Solvent: ethanol, brown crystals, yield 91%; mp. 207–209 °C; IR (KBr) ν [cm^−1^]: 3316, 3293, 3082, 3030, 2905, 1673, 1606, 1474, 1440, 1295, 1267, 1168, 1117, 1030, 796, 746, 736; Anal. Calcd. for C_9_H_11_Cl_2_CuN_3_O_2_ (327.65): C, 32.99; H, 3.38; N, 12.82; Found: C, 32.83; H, 3.34; N, 13.16.

Crystal data for **6a** CCDC no. 986200: C_9_H_11_Cl_2_CuN_3_O_2_, *M* = 327.65, triclinic, space group *P*-1 (no. 2), *Z* = 2, *a* = 7.3569(8) Å, *b* = 8.7648(8) Å, *c* = 9.7086(10) Å, α = 86.242(8), β = 86.557(9), γ = 77.925(9), *V* = 610.17(11) Å^3^, *T* = 293 K, μ(CuKα) = 6.521 mm^−1^, 7294 reflections measured, 2226 unique (*R*_int_ = 0.0366) which were used in all calculations. The final *wR*_2_ was 0.1024 (all data) and *R*_1_ was 0.0342 (I >2 σ (I)).

*Dichloro[1-(6-ethoxy-2-pyridyl)imidazolidin-2-one]copper(II)* (**6b**). Solvent: ethanol, brown crystals, yield 23%; mp. 199–202 °C; IR (KBr) ν [cm^−1^]: 3329, 3079, 2982, 2925, 1670, 1605, 1480, 1466, 1451, 1429, 1294, 1264, 1164, 1118, 1034, 1017, 786; Anal. Calcd. for C_10_H_13_Cl_2_CuN_3_O_2_ (341.68): C, 35.15; H, 3.83; N, 12.30; Found: C, 35.02; H, 3.79; N, 12.63.

*Dichloro[1-(6-n-propoxy-2-pyridyl)imidazolidin-2-one]copper(II)* (**6c**). Solvent: ethanol, brown crystals, yield 68%; mp. 185–187 °C; IR (KBr) ν [cm^−1^]: 3249, 3094, 2958, 2925, 2877, 1712, 1676, 1605, 1474, 1445, 1425, 1294, 1266, 1165, 1113, 1091, 993, 962, 792, 735; Anal. Calcd. for C_11_H_15_Cl_2_CuN_3_O_2_ (355.71): C, 37.14; H, 4.25; N, 11.81; Found: C, 37.10; H, 4.08; N, 12.08.

*Dichloro[1-(6-isopropoxy-2-pyridyl)imidazolidin-2-one]copper(II)* (**6d**). Solvent: ethanol, brown crystals, yield 56%; mp. 211–212 °C; IR (KBr) ν [cm^−1^]: 3303, 3112, 3056, 2983, 2931, 1672, 1603, 1469, 1441, 1425, 1372, 1293, 1266, 1166, 1115, 1092, 987, 952, 798, 734; Anal. Calcd. for C_11_H_15_Cl_2_CuN_3_O_2_ (355.71): C, 37.14; H, 4.25; N, 11.81; Found: C, 36.98; H, 4.18; N, 11.80.

*Dichloro[1-(6-n-butoxy-2-pyridyl)imidazolidin-2-one]copper(II)* (**6e**). Solvent: ethanol, golden crystals, yield 48%; mp. 187–189 °C; IR (KBr) ν [cm^−1^]: 3229, 3104, 2956, 2871, 1678, 1607, 1467, 1444, 1428, 1294, 1294, 1265, 1166, 1094, 790, 735; Anal. Calcd. for C_12_H_17_Cl_2_CuN_3_O_2_ (369.73): C, 38.98; H, 4.63; N, 11.36; Found: C, 38.91; H, 4.56; N, 11.62.

*Dichloro[1-(6-methoxy-2-pyridyl)imidazolidine-2-thione]copper(II)* (**6f**). Solvent: ethanol, brown crystals, yield 49%; mp. 149–154 °C; IR (KBr) ν [cm^−1^]: 3159, 3066, 2953, 2923, 1604, 1543, 1468, 1435, 1418, 1286, 1232, 1129, 787; Anal. Calcd. for C_9_H_11_Cl_2_CuN_3_OS (343.72): C, 31.45; H, 3.23; N, 12.23; Found: C: 31.38; H, 3.19; N, 11.88.

*Dichloro[1,3-bis(4-methyl-2-pyridyl)imidazolidin-2-one]copper(II)^.^H_2_O* (**7**). Solvent: methanol, green crystals; mp. 170–172°C; IR (KBr) ν [cm^−1^]: 3372, 1635, 1506, 1472, 1436, 1335, 1261, 1195.

Crystal data for **7** CCDC no. 986194: C_15_H_18_Cl_2_CuN_4_O_2_, *M* = 420.77, triclinic, space group *P*-1 (no. 2), *Z* = 2, *a* = 8.9310(7) Å, *b* = 10.6693(7) Å, *c* = 10.9789(9) Å, α = 112.749(7), β = 95.298(7), γ = 111.370(7), *V* = 864.67(11) Å^3^, *T* = 293 K, μ(MoKα) = 1.587 mm^−1^, 7039 reflections measured, 3517 unique (*R*_int_ = 0.0181) which were used in all calculations. The final *wR*_2_ was 0.0742 (all data) and *R*_1_ was 0.0287 (I > 2σ (I)).

*Dichloro[1,3-bis(2-pyridyl)imidazolidin-2-one]copper(II)* (**8a**). Solvent: ethanol, yellow-green crystals, yield 77%; mp. 265–267 °C; IR (KBr) ν [cm^−1^]: 3074, 2901, 1679, 1604, 1587, 1463, 1438, 1412, 1351, 1312, 1245, 1138, 781, 752, 740; Anal. Calcd. for C_13_H_12_Cl_2_CuN_4_O (374.71): C, 41.67; H, 3.23; N, 14.95; Found: C, 41.64; H, 3.19; N, 14.92.

Crystal data for **8a** CCDC no. 986095: C_26_H_24_Cl_4_Cu_2_N_8_O_2_, *M* = 749.43, monoclinic, space group *P*2_1_/n (no. 14), *Z* = 2, *a* = 7.83450(10) Å, *b* = 16.3329(2) Å, *c* = 10.8358(2) Å, β = 90.7160(10), *V* = 1386.44(4) Å^3^, *T* = 130 K, μ(MoKα) = 1.963 mm^−1^, 22938 reflections measured, 3444 unique (*R*_int_ = 0.0379) which were used in all calculations. The final *wR*_2_ was 0.0665 (all data) and *R*_1_ was 0.0293 [I > 2σ (I)].

*Dichloro[1-acetyl-3-(5-methyl-2-pyridyl)imidazolidin-2-one]copper(II)* (**8b**). Solvent: ethanol, green crystals, yield 50%; mp. 233–235 °C; IR (KBr) ν [cm^−1^]: 3091, 2984, 2927, 1698, 1655, 1468, 1434, 1401, 1323, 1291, 1256, 1042, 962, 839, 740, 617; Anal. Calcd. for C_11_H_13_Cl_2_CuN_3_O_2_ (353.69): C, 37.35; H, 3.70; N, 11.88; Found: C, 37.28; H, 3.64; N, 12.18.

*Dichloro[1-acetyl-3-(4-methyl-2-pyridyl)imidazolidin-2-one]copper(II)* (**8c**). Solvent: ethanol, green crystals, yield 62%; mp. 222–225 °C; IR (KBr) ν [cm^−1^]: 3091, 3060, 2986, 1708, 1683, 1622, 1455, 1412, 1372, 1321, 1271, 1192, 1150, 1038, 971, 841, 749, 742, 619, 456; Anal. Calcd. for C_11_H_13_Cl_2_CuN_3_O_2_ (353.69): C, 37.35; H, 3.70; N, 11.88; Found: C, 37.27; H, 3.63; N, 12.22.

Crystal data for **8c** CCDC no. 986202: C_22_H_26_Cl_4_Cu_2_N_6_O_4_, *M* = 707.39, triclinic, space group *P*-1 (no. 2), *Z* = 1, *a* = 8.6259(2) Å, *b* = 9.2649(3) Å, *c* = 10.4228(3) Å, α = 102.178(3), β = 98.752(2), γ = 116.880(3), *V* = 696.37(3) Å^3^, *T* = 293 K, μ(Cu Kα) = 5.765 mm^−1^, 7991 reflections measured, 2869 unique (*R*_int_ = 0.0137) which were used in all calculations. The final *wR*_2_ was 0.0738 (all data) and *R*_1_ was 0.0268 (I > 2σ (I)).

*Dichloro[1-butyryl-3-(4-methyl-2-pyridyl)imidazolidin-2-one]copper(II)* (**8d**). Solvent: ethanol, green crystals, yield 93%; mp. 236–240 °C; IR (KBr) ν [cm^−1^]: 3123, 3089, 3054, 2960, 2936, 2877, 1705, 1681, 1622, 1455, 1413, 1384, 1322, 1272, 1227, 1211, 1180, 907, 840, 743, 709, 663, 458; Anal. Calcd. for C_13_H_17_Cl_2_CuN_3_O_2_ (381.74): C, 40.90; H, 4.49; N, 11.01; Found: C, 40.82; H, 4.42; N, 10.98.

*Dichloro[1-acetyl-3-(4-tert-butyl-2-pyridyl)imidazolidin-2-one]copper(II)* (**8e**). Solvent: ethanol, green crystals, yield 53%; mp. 150–155 °C; IR (KBr) ν [cm^−1^]: 2966, 1662, 1619, 1478, 1445, 1377, 1283, 1063, 734, 617; Anal. Calcd. for C_14_H_19_Cl_2_CuN_3_O_2_ (395.77): C, 42.49; H, 4.84; N, 10.62; Found: C, 42.38; H, 4.82; N, 10.31.

*Dichloro[1-acetyl-3-(4-phenyl-2-pyridyl)imidazolidin-2-one]copper(II)* (**8f**). Solvent: ethanol, green crystals, yield 95%; mp. 227–233 °C; IR (KBr) ν [cm^−1^]: 3055, 2962, 2926, 1718, 1673, 1615, 1473, 1436, 1410, 1372, 1278, 1237, 965, 771, 740, 630, 615; Anal. Calcd. for C_16_H_15_Cl_2_CuN_3_O_2_ (415.76): C, 46.22; H, 3.64; N, 10.11; Found: C, 46.01; H, 3.61; N, 10.02.

*Dichloro[1-butyryl-3-(4-phenyl-2-pyridyl)imidazolidin-2-one]copper(II)* (**8g**). Solvent: ethanol, dark green crystals, yield 77%; mp. 178–184 °C; IR (KBr) ν [cm^−1^]: 3051, 2963, 2873, 1671, 1616, 1474, 1437, 1409, 1372, 1284, 1222, 1178, 768, 629; Anal. Calcd. for C_18_H_19_Cl_2_CuN_3_O_2_ (443.81): C, 48.71; H, 4.32; N, 9.47; Found: C, 48.68; H, 4.26; N, 9.45.

*Dichloro{1-acetyl-3-[4-(3-phenylpropyl)-2-pyridyl]imidazolidin-2-one}copper(II)* (**8h**). Solvent: ethanol, dark green crystals, yield 56%; mp. 160–164 °C; IR (KBr) ν [cm^−1^]: 3078, 2920, 2860, 1713, 1671, 1649, 1623, 1476, 1451, 1420, 1375, 1282, 1245, 966, 735, 701, 615; Anal. Calcd. for C_19_H_21_Cl_2_CuN_3_O_2_ (457.84): C, 49.84; H, 4.62; N, 9.18; Found: C, 49.79; H, 4.58; N, 9.46.

*Dichloro[1-acetyl-3-(4-methoxy-2-pyridyl)imidazolidin-2-one]copper(II)* (**8i**). Solvent: ethanol, light green crystals, yield 69%; mp. 190–194 °C; IR (KBr) ν [cm^−1^]: 3143, 3117, 3085, 3030, 2993, 2929, 1709, 1667, 1617, 1477, 146, 1377, 1281, 1232, 1037, 968, 845, 741, 616; Anal. Calcd. for C_11_H_13_Cl_2_CuN_3_O_3_ (369.69): C, 35.74; H, 3.54; N, 11.37; Found: C, 35.69; H, 3.46; N, 11.38.

*Dichloro[1-butyryl-3-(4-methoxy-2-pyridyl)imidazolidin-2-one]copper(II)* (**8j**). Solvent: ethanol, green crystals, yield 66%; mp. 210–213 °C; IR (KBr): 3104, 2967, 2930, 2873, 1671, 1618, 1468, 1419, 1382, 1285, 1220, 1176, 1048, 842, 738, 708; Anal. Calcd. for C_13_H_17_Cl_2_CuN_3_O_3_ (397.74): C, 39.26; H, 4.31; N, 10.56; Found: C, 39.11; H, 4.26; N, 10.35.

*Dichloro[1-acetyl-3-(4-benzyloxy-2-pyridyl)imidazolidin-2-one]copper(II)* (**8k**). Solvent: ethanol, green crystals, yield 49%; mp. 195–199 °C; IR (KBr) ν [cm^−1^]: 3086, 2990, 2923, 1683, 1613, 1480, 1448, 1415, 1378, 1278, 1245, 1036, 1022, 834, 738, 622; Anal. Calcd. for C_17_H_17_Cl_2_CuN_3_O_3_ (445.79): C, 45.80; H, 3.84; N, 9.43; Found: C, 45.71; H, 3.76; N, 9.76.

*Dichloro{bis[1-(5-methyl-2-pyridyl)imidazolidin-2-one]}copper(II)^.^2H_2_O* (**9**). 1-(5-Methyl-2-pyridyl)imidazolidin-2-one (0.1 g; 0.00056 mol) was dissolved in 7 mL of *N*,*N*-dimethylformamide and copper(II) chloride (0.144 g, 0.00085 mol) was added. After week of slow evaporation at room temperature green crystals suitable for the X-ray analysis were collected, washed with small amount of solvent and dried. Obtained 0.06 g of complex compound **9**, yield 43%: C_18_H_22_Cl_2_CuN_6_O_2_**^.^**2H_2_O (524.89); mp. 224–229 °C; IR (KBr) ν [cm^−1^]: 3169, 3068, 2915, 1657, 1618, 1576, 1483, 1453, 1319, 1287, 1170, 824, 742.

Crystal data for **9** CCDC no. 986199: (C_18_H_22_CuN_6_O_2_)Cl_2_·2(H_2_O) , *M* = 524.89, monoclinic, space group *C*2/c (no. 15), *Z* = 4, *a* = 12.3708(7) Å, *b* = 13.6659(5) Å, *c* = 13.1220(16) Å, β = 109.417(1), *V* = 2092.2(3) Å^3^, *T* = 130 K, μ(MoKα) = 1.340 mm^−1^, 7717 reflections measured, 1832 unique (*R*_int_ = 0.0450) which were used in all calculations. The final *wR*_2_ was 0.0803 (all data) and *R*_1_ was 0.0321 (I > 2σ (I)).

*Dichloro{bis[1-(4-phenyl-2-pyridyl)imidazolidin-2-one]}copper(II)* (**10a**, **10b**). 1-(4-Phenyl-2-pyridyl)imidazolidin-2-one (0.1 g, 0.00042 mol) was dissolved in 7 mL of *N*,*N*-dimethylformamide and copper(II) chloride (0.107 g, 0.00063 mol) was added. After two weeks of slow evaporation at room temperature green crystals suitable for the X-ray analysis were collected, washed with small amount of solvent and dried. Obtained 0.06 g of a mixture of complex compounds **10a** and **10b**, mp. 208–211 °C; IR (KBr) ν [cm^−1^]: 3447, 3056, 1669, 1616, 1473, 1446, 1319, 1296, 1070, 1014, 855, 763, 740.

Crystal data for **10a** CCDC no. 986198: (C_28_H_26_ClCuN_6_O_2_)Cl·2(H_2_O), *M* = 649.02, triclinic, space group *P*-1 (no. 2), *Z* = 2, *a* = 10.5234(4) Å, *b* = 11.7728(5) Å, *c* = 12.5141(4) Å, α = 97.231(3), β = 106.598(3), γ = 108.593(4), *V* = 1367.46(9) Å^3^, *T* = 100 K, μ(MoKα) = 1.042 mm^−1^, 14433 reflections measured, 5552 unique (*R*_int_ = 0.0169) which were used in all calculations. The final *wR*_2_ was 0.0695 (all data) and *R*_1_ was 0.0263 (I > 2σ (I)).

Crystal data for **10b** CCDC no. 986197: (C_28_H_26_ClCuN_6_O_2_)_2_[CuCl_3_]_0.75_[Cl]_0.5_P· 2.15(H_2_O)·C_3_H_7_NO, *M* = 1413.85, monoclinic, space group *I*2/a (no. 15), *Z* = 4, *a* = 13.8269(3) Å, *b* = 16.8610(3) Å, *c* = 25.7704(5) Å, β = 93.988(2), *V* = 5993.4(2) Å^3^, *T* = 100 K, μ(MoKα) = 1.246 mm^−1^, 34078 reflections measured, 6096 unique (*R*_int_ = 0.0240) which were used in all calculations. The final *wR*_2_ was 0.0827 (all data) and *R*_1_ was 0.0297 (I > 2σ (I)).The [CuCl_3_]^2−^ anion and one of the water molecules are located on a twofold axis. The Cu^I^ and the Cl atom of the anion in special position occupy their positions in 75% whereas the water molecule in 25%. The remaining Cl atoms fully occupy their positions.

*Dichloro{bis[1-(4-methoxy-2-pyridyl)imidazolidin-2-one]}copper(II)^.^H_2_O* (**10c**). 1-(4-Methoxy-2-pyridyl)imidazolidin-2-one (0.1 g; 0.00052 mol) was dissolved in 7 mL of *N*,*N*-dimethylformamide and copper(II) chloride (0.132 g, 0.00078 mol) was added. After two weeks of slow evaporation at room temperature blue crystals suitable for the X-ray analysis were collected, washed with small amount of solvent and dried. Obtained 0.05 g of complex compound **10c**, yield 36%: C_18_H_22_Cl_2_CuN_6_O_4_^.^H_2_O (538.87); mp. 214–216 °C; IR (KBr) ν [cm^−1^]: 3431, 3176, 2985, 1670, 1615, 1472, 1452, 1428, 1291, 1248, 1064, 1030.

Crystal data for **10c** CCDC no. 986195: (C_18_H_22_ClCuN_6_O_4_)Cl^.^H_2_O, *M* = 538.87, monoclinic, space group *P*2_1_ (no. 4), *Z* = 4, *a* = 10.9160(1) Å, *b* = 13.4955(2) Å, *c* = 15.1544(2) Å, β = 107.429(2), *V* = 2130.00(5) Å^3^, *T* = 120 K, μ(MoKα) = 1.322 mm^−1^, 36883 reflections measured, 9877 unique (*R*_int_ = 0.0225) which were used in all calculations. The final *wR*_2_ was 0.0576 (all data) and *R*_1_ was 0.0227 (I > 2σ (I)).

## 4. Conclusions

The X-ray crystallography revealed that the 1-(2-pyridyl)imidazolidin-2-ones **1**–**3** and 1-(2-pyridyl)imidazolidine-2-thiones **4** behaved as neutral bidentate ligands, bonding to the copper(II) ion through the nitrogen atom of pyridine ring and oxygen or sulfur atom of imidazolidin-2-one(thione) moiety. Analysis of the structure–activity relationships of anticancer activities of the diverse complexes **5**-**10** revealed that the most active against a panel of 5 human tumor cell lines was dichloro[1-(4-*tert*-butyl-2-pyridyl)imidazolidine-2-thione]copper(II) (**5j**), which may act as a “self-activating” chemical nuclease, and therefore, may serve as a lead structure for further development of novel anticancer agents.

## References

[1] Tapiero H., Townsend D.M., Tew K.D. (2003). Trace elements in human physiology and pathology. Copper. Biomed. Pharmacother..

[2] Tisato F., Marzano C., Porchia M., Pellei M., Santini C. (2010). Copper in diseases and treatments, and copper-based anticancer strategies. Med. Res. Rev..

[3] Chen J., Huang Y., Liu G., Afrasiabi Z., Sinn E., Padhye S., Ma Y. (2004). The cytotoxicity and mechanism of 1,2-naphthoquinone thiosemicarbazone and its metal derivatives against MCF-7 human breast cancer cells. Toxicol. Appl. Pharmacol..

[4] Easmon J., Pürstinger G., Heinisch G., Roth T., Fiebig T.R., Holzer W., Jäger W., Jenny M., Hofmann J. (2001). Synthesis, cytotoxicity, and antitumor activity of copper(II) and iron(II) complexes of ^4^*N*-azabicyclo[3.2.2]nonane thiosemicarbazones derived from acyl diazines. J. Med. Chem..

[5] Adsule S., Barve V., Chen D., Ahmed F., Dou Q.P., Padhye S., Sarkar F.H. (2006). Novel Schiff base copper complexes of quinoline-2 carboxaldehyde as proteasome inhibitors in human prostate cancer cells. J. Med. Chem..

[6] Marzano C., Pellei M., Colavito D., Alidori S., Lobbia G.G., Gandin V., Tisato F., Santini C. (2006). Synthesis, characterization, and* in vitro* antitumor properties of tris(hydroxymethyl)phosphine copper(I) complexes containing the new bis(1,2,4-triazol-1-yl)acetate ligand. J. Med. Chem..

[7] Tardito S., Bussolati O., Maffini M., Tegoni M., Giannetto M., Dall’Asta V., Franchi-Gazzola R., Lanfranchi M., Pellinghelli M.A., Mucchino C. (2007). Thioamido coordination in a thioxo-1,2,4-triazole copper(II) complex enhances nonapoptotic programmed cell death associated with copper accumulation and oxidative stress in human cancer cells. J. Med. Chem..

[8] Sączewski F., Dziemidowicz-Borys E., Bednarski P.J., Gdaniec M. (2007). Synthesis, crystal structure, cytotoxic and superoxide dismutase activities of copper(II) complexes of *N*-(4,5-dihydroimidazol-2-yl)azoles. Arch. Pharm. Chem. Life Sci..

[9] Marzano C., Pellei M., Tisato F., Santini C. (2009). Copper complexes as anticancer agents. Anticancer Agents Med. Chem..

[10] Santini C., Pellei M., Gandin V., Porchia M., Tisato F. (2014). Advances in copper complexes as anticancer agents. Chem. Rev..

[11] Sączewski F., Dziemidowicz-Borys E., Bednarski P.J., Grünert R., Gdaniec M., Tabin P. (2006). Synthesis, crystal structure and biological activities of copper(II) complexes with chelating bidentate 2-substituted benzimidazole ligands. J. Inorg. Biochem..

[12] Szilágyi I., Labádi I., Hernadi K., Pálinko I., Kiss T. (2005). Synthesis and IR spectroscopic characterization of immobilized superoxide dismutase (SOD) mimicking complexes. J. Mol. Struct..

[13] Devereux M., O’Shea D., O’Connor M., Grehan H., Connor G., McCann M., Rosair G., Lyng F., Kellet A., Walsh M. (2007). Synthesis, catalase, superoxide dismuthase and antitumour activities of copper(II) carboxylate complexes incorporating benzimidazole, 1,10-phenanthroline and bipyridine ligands: X-ray crystal structures of [Cu(BZA)_2_(bipy)(H_2_O)], [Cu(SalH)_2_(BZDH)_2_] and [Cu(CH_3_COO)_2_(5,6-DMBZDH)_2_] (SalH_2_ = salicylic acid; BZAH = benzoic acid; BZDH = benzimidazole and 5,6-DMBZDH = 5,6-dimethylbenzimidazole). Polyhedron.

[14] Mendes I.C., Moreira J.P., Mangrich A.S., Balena S.P., Rodrigues B.L., Beraldo H. (2007). Coordination to copper(II) strongly enhances the* in vitro* antimicrobial activity of pyridine-derived *N*(4)-tolyl thiosemicarbazones. Polyhedron.

[15] Sharma S., Athar F., Maurya M.R., Naqvi F., Azam A. (2005). Novel bidentate complexes of Cu(II) derived from 5-nitrofuran-2-carboxaldehyde thiosemicarbazones with antiamoebic activity against *E. histolytica*. Eur. J. Med. Chem..

[16] Weder J.E., Dillon C.T., Hambley T.W., Kennedy B.J., Lay P.A., Biffin J.R., Regtop H.L., Davies N.M. (2002). Copper complexes of non-steroidal anti-inflammatory drugs: An opportunity yet to be realized. Coord. Chem. Rev..

[17] Gennäs G.B., Mologni L., Ahmed S., Rajaratnam M., Marin O., Lindholm N., Viltadi M., Gambacorti-Passerini C., Scapozza L., Yli-Kauhaluoma J. (2011). Design, synthesis, and biological activity of urea derivatives as anaplastic lymphoma kinase inhibitors. Chem. Med. Chem..

[18] Koca I., Özgür A., Coşkun K.A., Tutar Y. (2013). Synthesis and anticancer activity of acyl thioureas bearing pyrazole moiety. Bioorg. Med. Chem..

[19] Petitclerc É., Deschesnes R.G., Côté M.F., Marquis C., Janvier R., Lacroix J., Miot-Noirault É., Legault J., Mounetou E., Madelmont J.C. (2004). Antiangiogenic and antitumoral activity of phenyl-3-(2-chloroethyl)ureas: A class of soft alkylating agents disrupting microtubules that are unaffected by cell adhesion-mediated drug resistance. Cancer Res..

[20] Stabile P., Lamonica A., Ribecai A., Castoldi D., Guercio G., Curcuruto O. (2010). Mild, convenient and versatile Cu-mediated synthesis of *N*-aryl-2-imidazolidinones. Tetrahedron Lett..

[21] Sączewski F., Bułakowska A., Gdaniec M. (2002). 2-Chloro-4,5-dihydroimidazole, Part X. revisiting route to *N*-heteroarylimidazolidin-2-ones. J. Heterocycl. Chem..

[22] Balewski Ł., Sączewski F., Gdaniec M., Bednarski P.J., Jara I. (2013). Synthesis of *N*-(2-pyridyl)imidazolidin-2-ones and 1-(2-pyridyl)-2,3,7,8-tetrahydro-1*H*-imidazo[2,1-*b*][1,3,5]-triazepin-5(6*H*)-ones with potential biological activity. Heterocycl. Commun..

[23] Venkataraman D., Du Y., Wilson S.R., Hirsch K.A., Zhang P., Moore J.S. (1997). A coordination geometry table of the *d*-block elements and their ions. J. Chem. Educ..

[24] Dolomanov O.V., Bourhis L.J., Gildea R.J., Howard J.A.K., Puschmann H. (2009). OLEX2: A complete structure solution, refinement and analysis program. J. Appl. Cryst..

[25] Tiliakos M., Cordopatis P., Terzis A., Raptopoulou C.P., Perlepes S.P., Manessi-Zoupa E. (2001). Reactions of 3d-metal nitrates with *N*,*N*'-bis(2-pyridyl)urea (LH_2_): preparation, X-ray crystal structures and spectroscopic studies of the products *trans*-[M(II)(ONO_2_)_2_(LH_2_)_2_] (M = Mn, Fe, Co, Ni, Cu, Zn) and *mer*-[Co(III)(LH)_2_](NO_3_)**^.^**MeOH. Polyhedron.

[26] Bracht K., Boubakari, Grünert R., Bednarski J.P. (2006). Correlations between the activities of 19 antitumor agents and the intracellular glutatione concentrations in a panel of 14 human cancer cell lines: comparisons with the National Cancer Institute data. Anticancer Drugs.

[27] Ghosh K., Kumar P., Mohan V., Singh U.P., Kasiri S. (2012). Nuclease activity via self-activation and anticancer activity of a mononuclear copper(II) complex: Novel role of the tertiary butyl group in the ligand frame. Inorg. Chem..

[28] Li L., Du K., Wang Y., Jia H., Hou X., Chao H., Ji L. (2013). Self-activating nuclease and anticancer activities of copper(II) complexes with aryl-modified 2,6-di(thiazol-2-yl)pyridine. Dalton Trans..

[29] Li K., Zhou L.-H., Zhang J., Chen S.-Y., Zhang Z.-W., Zhang J.-J., Lin H.-H., Yu X.-Q. (2009). “Self-activating” chemical nuclease: Ferrocenyl cyclen Cu(II) complexes act as efficient DNA cleavage reagents in the absence of reductant. Eur. J. Med. Chem..

[30] (2009). CrysAlis Software.

[31] Sheldrick G.M. (2008). A short history of SHELX. Acta Crystallogr..

